# Perspectives on interpersonal touch are related to subjective sleep quality

**DOI:** 10.1111/jsr.13766

**Published:** 2022-11-09

**Authors:** Anna Lena Dueren, Natalie C. Bowling, Aikaterini Vafeiadou, Juan J. Madrid‐Valero, Claudia Hammond, Alice M. Gregory, Michael J. Banissy

**Affiliations:** ^1^ Department of Psychology, Goldsmiths University of London London UK; ^2^ School of Human Sciences University of Greenwich London UK; ^3^ Department of Health Psychology, Faculty of Health Science University of Alicante Alicante Spain; ^4^ School of Psychology University of Sussex Brighton UK; ^5^ School of Psychological Science University of Bristol Bristol UK

**Keywords:** co‐sleep, c‐tactile, ct‐afferent, sleep, touch

## Abstract

Affective touch has been reported for its calming effects; however, it is less clear whether touch is associated with sleep. Here, the relationship between different touch variables and self‐reported sleep indicators was investigated. Data were extracted from the Touch Test, a cross‐sectional survey conducted in 2020. Data from a sample of 15,049 healthy adults from the UK (mean age = 56.13, SD = 13.8; 75.4% female) were analysed. Participants were asked to attribute positive, negative, or no effects on sleep to hugs, strokes, massages, intimate touch, and sleep onset with and without touch. The time since last intentional touch, touch amount satisfaction, and childhood bed routine with hugs and kisses were assessed. Sleep quality, duration, latency, wake after sleep onset and diurnal preference were measured. Data were analysed using chi‐square tests and logistic regressions. Affective touch before sleep was perceived to have positive effects on sleep. Touch recency emerged as a significant predictor for some sleep variables, with a longer timespan since the last intentional touch relating to improved sleep quality, longer sleep duration, and shorter and fewer instances of waking up after sleep onset in some participants. Experiencing too much touch was related to lower sleep quality and higher instances of waking up after sleep onset. These findings highlight the importance of interpersonal touch for subjective sleep quality.

## 1 INTRODUCTION

In Western society, cohabiting couples commonly share a bed (National Sleep Foundation, 2005). Co‐sleeping allows for partner touch before and during sleep onset as well as throughout the night (Hislop, 2007; Junker et al., 2016; Kirkman, 2010). Yet, the relationship between touch and sleep quality in adults is widely understudied (Dueren et al., 2021).

From a theoretical perspective, a relationship between affective touch and sleep quality might be expected because engagement in affective touch has consistently been linked to stress reduction (e.g., Eckstein et al., 2020; Field, 2019; Hesse et al., 2020; Morrison, 2016). Simultaneously, stressful events can contribute to disturbed sleep or even chronic insomnia (Basta et al., 2007; Kalmbach et al., 2018). Therefore, affective touch might be expected to enhance sleep quality by reducing stress. However, research in this area has focussed primarily on the relationship between sexual touch and sleep. In contrast, other types of pre‐sleep touch or touch throughout the day have rarely been researched in relation to sleep (Dueren et al., 2021). Even retrospective research on the relationship between touch experiences during childhood and sleep outcomes in adulthood primarily assessed how sexual abuse in childhood relates to disturbed sleep patterns in adulthood (Kajeepeta et al., 2015; Steine et al., 2012).

Additionally, individual differences should be considered to understand possible relationships between touch and sleep, as they might contribute to individual differences in sleep outcomes. Firstly, gender likely contributes to individual differences in sleep variables, as gender‐dependent differences have been reported previously in relation to sleep and the impact of touch on sleep (Dittami et al., 2007; Lastella et al., 2019; Pallesen et al., 2020; Sa et al., 2020). Next, attachment style has consistently been associated with differences in touch experience (e.g., Adams et al., 2014; Beltrán et al., 2020; Chopik et al., 2014; Jakubiak & Feeney, 2017; Kim et al., 2018; Krahé et al., 2016, 2018; Wagner et al., 2020). Lastly, previous research has linked loneliness to sleep disturbances (Griffin et al., 2020). Hence, these variables were included in the present analyses.

Here, we aimed to extend the existing literature about the relationship between touch and sleep. We analyse (1) how various types of pre‐sleep affective touch are judged to influence sleep and (2) whether a range of touch experiences are associated with sleep outcomes while considering relevant interpersonal differences. Specifically, for (2), we investigate whether touch amount satisfaction (i.e., whether a person is content with the amount of touch in their life), touch recency (i.e., when a person was last touched intentionally), and retrospectively assessed childhood touch experiences at bedtime relate to individual differences in sleep outcomes. Analyses were conducted by drawing on data from an extensive cross‐sectional survey on topics surrounding touch, the Touch Test (described below). The following hypotheses were formulated:

Hypothesis 1 : Falling asleep whilst touching a partner and pre‐sleep hugging, massaging, stroking, and intimate touch will be judged to have more positive than negative or neutral effects on sleep quality as assessed by touch and sleep judgments.

Hypothesis 2 : Touch recency and touch amount satisfaction will be associated with sleep variables.

Hypothesis 3 : Respondents who reported more childhood hugs and kisses at bedtime would report better sleep outcomes than those who reported fewer childhood hugs and kisses.

Hypothesis 4 : Age, gender, loneliness, and attachment style may contribute to the relationship between interpersonal touch and sleep.

## 2 PARTICIPANTS AND METHODS

### 2.1 Data collection

Data for this study were drawn from the Touch Test, a survey conducted as part of a science communication project that explored various aspects of touch attitudes and behaviours in a worldwide sample. Data for the Touch Test were collected between 21 January 2020 and 30 March 2020. Participation was voluntary, and the participants were not compensated for participation. The study was conducted online; participants completed the questionnaire within 7 days of starting the survey. The participants gave informed consent before completing the questionnaire. The local university ethical committee approved the study.

### 2.2 Study participants

The participants were recruited through promotions on BBC radio, TV programmes, and other media. To participate, the respondents had to be at least 18 years old and have internet access. Per pre‐registered analysis plans, the analysis reported here was restricted to healthy participants by excluding data of respondents who reported current disabilities, long‐term condition(s), or impairment(s). Further, only male and female UK adults were included in this analysis. The decision to include only UK adults was taken because there are substantial differences in touch perception across cultures (Gallace & Spence, 2010). While the full Touch Test sample included participants from 113 countries, the UK was the largest cohort, and there were large differences in group sizes between countries (e.g., for some countries *N* = 1 responder). The UK was the only country with over 1000 responders, and power analysis indicated that at least 1621 respondents would be needed to detect a small effect size of *f* ^*2*^ = 0.02 at 0.01 alpha error probability and 0.95 power with 11 predictors (note that two of the predictors were ultimately not used, as explained in the section “Deviations from pre‐registered analysis” below). Therefore, it was decided at the pre‐registration stage that analyses would focus on the UK only to provide the statistical power required to give a more reliable population‐based estimate. The final sample analysed for the current report consisted of 15,049 healthy adults from the UK. The participants were aged 18–92 years (M = 56.13, SD = 13.8, 75.4% female). Note that the sample number slightly varies across analyses due to participant dropout and unanswered items in the survey. The *N* for each analysis is reported with the results.

### 2.3 Measures

The extracted variables from the Touch Test are presented below. For the complete Touch Test questionnaire, see our pre‐registration. The following demographic variables were analysed: age, gender, and survey completion date (hereafter end date). The variables were chosen because they allowed us to assess the relationship between sleep outcomes and a subjective judgement of touch experience (touch amount satisfaction) and to assess the relationship between sleep outcomes and a more objective measure of touch experience (touch recency).

The end date was included as a covariate in the present study because data collection fell within the expansion of the COVID‐19 pandemic in the UK, which might have affected opportunities to touch and attitudes towards touch.

*Touch and sleep judgements* were collected by asking participants to classify touch/bed‐sharing descriptions as positive, negative, or neutral for sleep quality. Participants could select one of these options for each touch descriptor. Descriptors were: “A gentle stroke by your partner”, “A short good‐night hug from your partner”, “A massage from your partner”, “Intimate touch from your partner”, “Preparing to sleep in a position in which you and your partner are touching one another”, and “Preparing to sleep in a position in which you and your partner are not touching one another”.

*Childhood bed routine* was assessed with an item taken from the Touch Experiences and Attitudes Questionnaire (TEAQ) (Trotter et al., 2018): “As a child, my parents would tuck me up in bed every night and give me a hug and a kiss good‐night”, answered on a 5‐point scale ranging from “Disagree strongly” to “Agree strongly”, with higher scores indicating stronger agreement.

*Diurnal preference* was assessed by one item derived from the morningness‐eveningness questionnaire (MEQ) (Horne & Oestberg, 1976): “One hears about ‘morning’ and ‘evening’ types of people. Which one of these do you consider yourself to be?” With four response options: “Definitely a morning type”, “rather more a morning type than an evening type”, “rather more an evening type than a morning type”, and “definitely an evening type”.

*Sleep variables* were measured by items adapted from the Pittsburgh Sleep Quality Index (PSQI) (Buysse et al., 1989). Variables were sleep quality, sleep duration, sleep latency, wake after sleep onset (WASO) duration, and frequency of WASO occurrences (WASO numbers). Sleep quality had four response options, ranging from “Very good” to “Very bad”, with higher scores indicating worse sleep quality. Sleep duration had five response options, ranging from “>9 h” to “<5 h”, with lower scores indicating longer sleep duration. Sleep latency, WASO duration, and WASO numbers had five response options, with higher numbers indicating longer sleep latency and WASO duration and higher WASO numbers. Sleep latency and WASO duration answer options ranged from “0–15 min” to “61 min or more”, WASO numbers answer options ranged from “0” to “4 or more”.

*Touch recency* was measured by one item: “When was the last time that somebody touched you intentionally, not including formal gestures such as handshakes in meetings?” answered on a 6‐point scale ranging from “In the last hour” to “Over a year ago” (higher scores indicate less recent touch).

*Touch amount satisfaction* was assessed by one item, “Thinking about the amount of touch in your life is it…”, answered on a 5‐point scale ranging from “Definitely too little” to “Definitely too much”. Note that both poles indicate dissatisfaction with the touch amount in one's life, while satisfaction was indicated by answer option 3, “just the right amount”. To distinguish between participants who experienced too much and those who experienced too little touch, the touch amount satisfaction item was separated into two subscales and rescored; one scale (hereafter referred to as the “Too little touch scale”), ranging from “Definitely too little” (scored as 1) to “Just the right amount” (scored as 3) and the second one (hereafter referred to as the “Too much touch scale”), ranging from “Definitely too much” (scored as 1) to “Just the right amount” (scored as 3). Higher scores thus indicate greater touch amount satisfaction in both scales. All regressions were performed twice; one with the too little touch scale and one with the too much touch scale.

*Attachment anxiety and avoidance* were measured by a 12‐item version of the Experiences in Close Relationships Questionnaire (ECR‐12) (Lafontaine et al., 2015). Two subscales indicate anxious and avoidant attachment styles, with higher scores indicating higher attachment anxiety or avoidance. Response options were measured on a 7‐point scale ranging from “Strongly disagree” to “Strongly agree”. The subscales were found to have good internal consistencies (α between 0.74 and 0.87 for both subscales), and adequate convergent validity as assessed by the relationship between ECR‐12 scores and psychological distress and relationship satisfaction scales (Lafontaine et al., 2015).

*Loneliness* was measured by the revised University of California Loneliness Scale (UCLA‐LSR) (Panayiotou et al., 2022; Russell et al., 1980), with higher scores indicating greater loneliness. Responses were measured on a continuous scale ranging from 0 to 100. The UCLA‐LSR has been found to possess good internal consistency (*α* = 0.94) and adequate concurrent validity as assessed by comparison with depression and anxiety scales (Russell et al., 1980).

### 2.4 Data analysis

#### 2.4.1 Hypothesis 1

Chi‐square goodness‐of‐fit tests were run to test whether the frequencies of each touch descriptor were equal across positive, neutral, and negative answer categories. Separate tests were run for each type of touch assessed. Bonferroni‐corrected binomial tests were run for post‐hoc comparisons comparing all pairs of answer possibilities (negative vs. neutral, positive vs. neutral, negative vs. positive) for each type of touch. Next, to assess whether there were gender differences in touch judgements, chi‐square tests of independence were run, comparing the answers of women and men. Separate analyses were run for each touch type, with adjusted residuals used for post‐hoc comparisons. Note also that a small proportion of participants (*N* = 65) indicated that they had technical problems completing the touch and sleep judgements. These participants were removed from the analyses for hypothesis 1.

#### 2.4.2 Hypotheses 2, 3 and 4

To evaluate the associations between touch amount satisfaction, touch recency, childhood bed routine, loneliness, attachment anxiety and avoidance, gender, age, and end date on sleep outcomes and diurnal preference, separate regression analyses were run for each outcome variable. Ordinal logistic regressions were run for sleep outcomes, and a multinomial logistic regression was conducted for diurnal preference. Additionally, linear and quadratic multiple regression models were compared for sleep duration. This was done because the initial linear model showed poor model fit, and from a theoretical perspective, both too short and too long sleep is suboptimal (Cappuccio et al., 2010; Jike et al., 2018). Hence, we were interested in testing whether a quadratic model would fit the data so that suboptimal sleep predictors (e.g., fewer reported instances of childhood bed routine, as per hypothesis 3) would be associated with very short and very long sleep durations. Statistical significance was set at *p* < 0.01 to account for the large sample size and avoid Type I error in hypothesis testing. For scales and subscales that consisted of several items (ECR‐12 and UCLA‐LSR), participants were included if they completed at least 80% of the items of the respective questionnaire.

#### 2.4.3 Deviations from pre‐registered analysis

There were no major deviations from the pre‐registered analysis for hypotheses 1 and 3. For hypothesis 4, we did not analyse sexual preference or ethnicity due to limited sample sizes in some of these groups restricting statistical power.

Sensitivity analyses were conducted to identify whether the results were consistent across groups of participants experiencing more recent touch and those who had not recently experienced touch. Participants were split according to their responses on the touch recency item. Scores of 1–3 (i.e., received touch in the last hour, the previous day or the last week) were combined into a “more recent touch” group (*N* = 13,840), and scores of 4–6 (i.e., received touch in the past month, over a month ago, or over a year ago) were combined in to “less recent touch” group (*N* = 1207). The overall pattern of results did not differ across groups, for full details see Supplementary Results.

During the review process, it was suggested that additional analyses on the possible moderating effects of attachment style on sleep outcomes might be beneficial. As such, exploratory analyses investigated whether attachment style moderates the relationship between touch recency, touch satisfaction, and sleep outcome variables. For this purpose, separate moderation analyses were run. Mean centring was used for the relevant variables (touch recency, touch satisfaction, avoidant attachment, and anxious attachment). Then, the four interaction terms (touch recency × avoidant attachment, touch recency × anxious attachment, touch satisfaction × avoidant attachment, touch satisfaction × anxious attachment) were calculated by multiplying the mean‐centred variables of a given interaction. This process was followed to avoid multicollinearity issues between the interaction terms.

## 3 RESULTS

### 3.1 Hypothesis 1: Self‐judged effects of partner touch during sleep onset and pre‐sleep hugging, massaging, stroking, and intimate touch on sleep quality

#### 3.1.1 Self‐judged effects of touch on sleep quality

**FIGURE 1:**
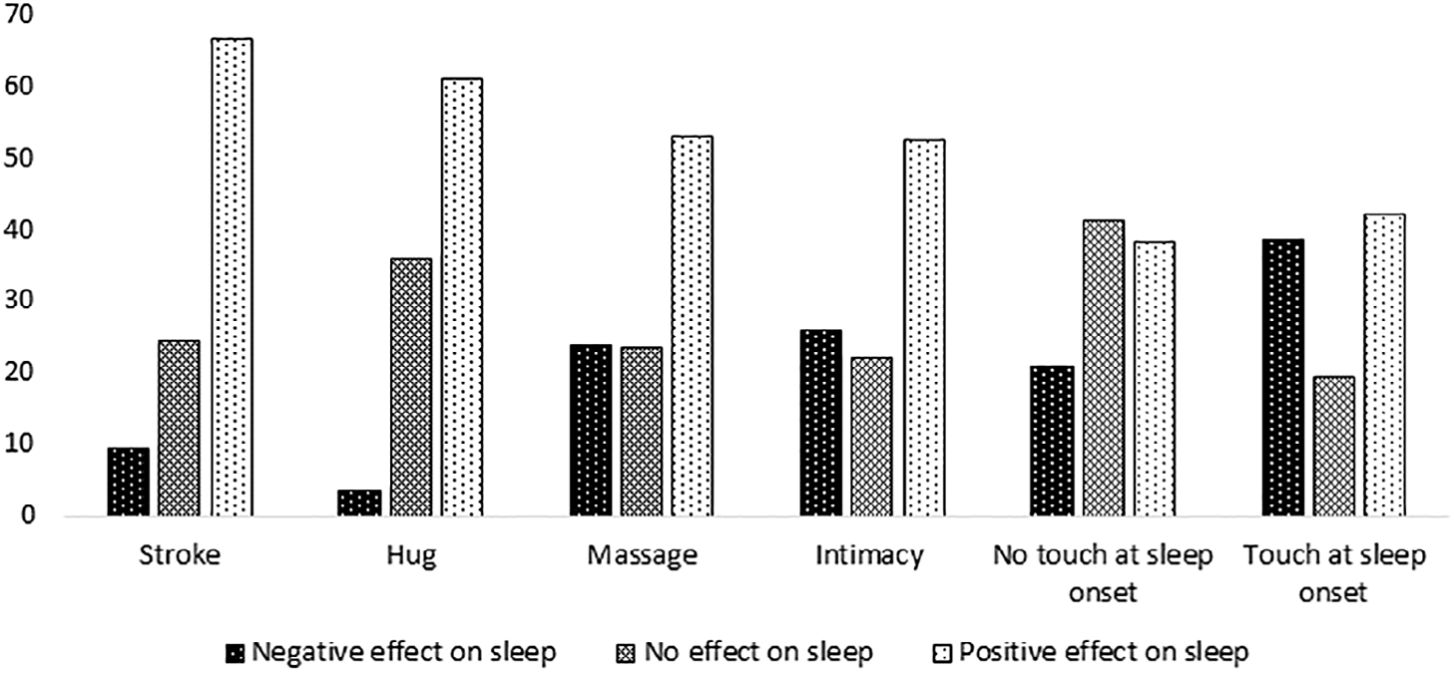
Touch judgements in percent. Statistics for the chi‐square goodness‐of‐fit tests assessing distribution across judgements were all significant at *p* < 0.001 with test statistics as followed: stroke *χ* ^2^(2) = 5435.411; hug *χ* ^2^(2) = 5218.573; massage *χ* ^2^(2) = 1775.379; intimacy *χ* ^2^(2) = 1655.387; no touch at sleep onset *χ* ^2^(2) = 762.423; touch at sleep onset *χ* ^2^(2) = 955.628

The chi‐square goodness‐of‐fit tests comparing the distributions of answers were statistically significant for each type of touch (Figure 1). This means that for each type of touch, the answer categories “negative effect on sleep”, “no effect on sleep”, and “positive effect on sleep” were not chosen with the same frequencies. Pairwise comparisons using binomial tests showed that most pairs of answer categories were chosen with significantly different frequencies (Table 1). These results were consistent with the hypothesis that affective touch would be perceived to enhance sleep rather than to hinder it. Partner touch during sleep onset was perceived to enhance sleep in some participants and to impair sleep in others (Table 1).

**TABLE 1:** Observed proportions in percent for pairwise comparisons with binomial tests

Comparison	Stroke	Hug	Massage	Intimacy	Not touching partner at sleep onset	Touching partner at sleep onset
Negative versus neutral						
Observed proportion						
Negative	28%	9%	50%	54%	34%	67%
Neutral	72%	91%	50%	46%	66%	33%
*p*	**<0.001**	**<0.001**	1.632	**<0.001**	**<0.001**	**<0.001**
Neutral versus positive						
Observed proportion						
Neutral	27%	37%	31%	30%	52%	32%
Positive	73%	63%	69%	70%	48%	68%
*p*	**<0.001**	**<0.001**	**<0.001**	**<0.001**	**0.002**	**<0.001**
Negative versus positive						
Observed proportion						
negative	12%	5%	31%	33%	35%	48%
positive	88%	95%	69%	67%	65%	52%
*p*	**<0.001**	**<0.001**	**<0.001**	**<0.001**	**<0.001**	**<0.001**
*N* per analysis	10,416	10,530	10,191	10,237	10,616	10,735

*Note*: Significance levels are Bonferroni corrected, hence bold fonts significance is set at 0.016 (0.05/3). Note that the answers given for the third category are not considered here to facilitate pairwise comparison; therefore observed proportions add up to 100%.

#### 3.1.2 Gender differences in judged effects of touch on sleep

Chi‐square tests of independence comparing touch judgements of women and men were conducted to assess whether women and men judged touch effects on sleep similarly. Results showed that women and men differed in their judgement of all types of touch, except for hugs (Tables 2 and 3). Adjusted residuals showed that women were less likely to attribute positive effects on sleep to strokes, massage, intimacy, and touch at sleep onset than men. Men were more likely to attribute positive effects on sleep to these types of touch than women. Correspondingly, women were more likely to attribute adverse effects on sleep to strokes, massage, intimacy, and touch at sleep onset. In contrast, men were less likely to attribute adverse effects to these types of touch.

**TABLE 2:** Chi‐square test of independence comparing touch judgements (for stroke, hug, massage, or intimacy) across men and women

Judgement	Stroke		Hug		Massage		Intimacy	
	Men	Women	Men	Women	Men	Women	Men	Women
Negative effect on sleep								
Percent responses	1.8%	7.7%	0.8%	2.6%	4.3%	19.4%	4.2%	21.6%
Adjusted residual	**−4.3**	**4.3**	−0.9	0.9	**−7.7**	**7.7**	**−11.4**	**11.4**
No effect on sleep								
Percent responses	5.6%	18.7%	8.7%	27.3%	6.1%	17.2%	4.2%	17.8%
Adjusted residual	−1.6	1.6	−0.2	0.2	2.8	−2.8	**−6.6**	**6.6**
Positive effect on sleep								
Percent responses	16.9%	49.4%	14.8%	45.9%	13.7%	39.3%	16.1%	36.1%
Adjusted residual	**4.1**	**−4.1**	0.5	−0.5	**4.2**	**−4.2**	**15.5**	**−15.5**
Chi‐square test of independence	χ^2^(2) = 23.952, *p* < 0.001		*χ* ^2^(2) = 0.892, *p =* 0.640		χ^2^(2) = 59.607, *p* < 0.001		χ^2^(2) = 245.247, *p* < 0.001	

*Note*: Adjusted residuals are considered to indicate a significant difference when they are < −3 or >3, hence bold fonts statistical significance set at *p* < 0.05.

**TABLE 3:** Chi‐square test of independence comparing touch judgements (for touch or no touch at sleep onset) across men and women

	No touch at sleep onset		Touch at sleep onset	
	Men	Women	Men	Women
Negative effect on sleep				
Percent responses	5.3%	15.5%	7.9%	30.7%
Adjusted residual	1.5	−1.5	**−7.1**	**7.1**
No effect on sleep				
Percent responses	11.5%	29.5%	5.0%	14.4%
Adjusted residual	**7.8**	**−7.8**	2.3	−2.3
Positive effect on sleep				
Percent responses	7.4%	30.7%	11.2%	30.8%
Adjusted residual	**−9.2**	**9.2**	**5.2**	**−5.2**
Chi‐square test of independence	χ^2^(2) = 90.443, *p* < 0.001		χ^2^(2) = 50.908, *p* < 0.001	

*Note*: Adjusted residuals are considered to indicate a significant difference when they are < −3 or >3, hence bold fonts statistical significance set at *p* < 0.05.

### 3.2 Hypotheses 2, 3 and 4: How are touch recency, touch amount satisfaction, childhood bed routine, age, gender, loneliness, and attachment style related to sleep variables?

Ordinal logistic regressions were used to assess sleep outcomes from touch amount satisfaction, touch recency, childhood bed routine, age, gender, attachment anxiety and avoidance, loneliness, and end date. Pearson and deviance chi‐square goodness‐of‐fit measures were nonsignificant, apart from the Pearson goodness‐of‐fit measures for sleep duration (with *p* = 0.009 for the too little touch scale analysis and *p* = 0.017 for the too much touch scale analysis), indicating a good model fit across most sleep outcomes.

#### 3.2.1 Hypothesis 2: Touch recency and touch amount satisfaction

Touch recency was significantly associated with sleep outcomes (Tables 4, 5, 6, 7, 8, 9). Specifically, a longer period following the most recent touch was related to better sleep quality and longer sleep duration in the too little touch amount satisfaction analyses. A longer time since last touch was associated with shorter and fewer WASOs; again this relationship reached statistical significance only for the analysis including the too little touch scale (Tables 7, 8, 9). Lastly, a multinomial logistic regression was used to assess the association between touch and diurnal preference (Table 10). With “Definitely an evening type” as the reference category, a longer time since last intentional touch was associated with a lower probability of indicating that one is more a morning type than an evening type.

**TABLE 4:** Ordinal logistic regression predicting sleep quality with too little to just right touch satisfaction

Predictor	OR	95% confidence interval for OR		SE	*χ* ^2^	*p*
		Lower	Upper			
Gender	0.910	0.838	0.989	0.042	4.965	0.026
Age	1.000	0.998	1.003	0.001	0.040	0.842
End date	0.996	0.994	0.998	0.001	16.788	**<0.001**
Loneliness	1.032	1.027	1.036	0.002	211.931	**<0.001**
Childhood bed routine	0.972	0.948	0.998	0.013	4.534	0.033
Touch recency	0.897	0.861	0.935	0.021	26.269	**<0.001**
Touch satisfaction – too little to just right	0.944	0.896	0.996	0.027	4.520	0.033
Attachment avoidance	1.005	0.999	1.010	0.003	3.018	0.082
Attachment anxiety	1.023	1.018	1.028	0.002	93.647	**<0.001**
Touch recency × Attachment avoidance	0.999	0.994	1.003	0.002	0.396	0.529
Touch recency × Attachment anxiety	1.001	0.996	1.005	0.002	0.137	0.712
Touch satisfaction × Attachment avoidance	1.007	1.000	1.014	0.003	4.282	0.039
Touch satisfaction × Attachment anxiety	1.000	0.994	1.006	0.003	0.017	0.895

*Note*: Lower scores indicate better sleep quality. For gender, women are the reference category. *N* = 11,625, hence bold fonts significance set at *p* < 0.01.

**TABLE 5:** Ordinal logistic regression predicting sleep quality with too much to just right touch satisfaction

Predictor	OR	95% confidence interval for OR		SE	*χ* ^2^	*p*
		Lower	Upper			
Gender	0.833	0.730	0.951	0.068	7.303	0.007
Age	0.999	0.995	1.003	0.002	0.283	0.595
End date	0.997	0.995	1.000	0.002	3.274	0.070
Loneliness	1.036	1.029	1.044	0.004	105.323	**<0.001**
Childhood bed routine	0.974	0.937	1.013	0.020	1.768	0.184
Touch recency	0.960	0.896	1.028	0.035	1.385	0.239
Touch satisfaction – too much to just right	0.679	0.556	0.828	0.102	14.540	**<0.001**
Attachment avoidance	1.007	0.999	1.015	0.004	2.591	0.107
Attachment anxiety	1.024	1.016	1.031	0.004	40.784	**<0.001**
Touch recency × Attachment avoidance	0.998	0.991	1.005	0.004	0.351	0.554
Touch recency × Attachment anxiety	1.005	0.998	1.012	0.004	1.728	0.189
Touch satisfaction × Attachment avoidance	1.003	0.983	1.024	0.010	0.093	0.761
Touch satisfaction × Attachment anxiety	0.981	0.961	1.002	0.011	3.277	0.070

*Note*: Lower scores indicate better sleep quality. For gender, women are the reference category. *N* = 5414, hence bold fonts significance set at *p* < 0.01.

**TABLE 6:** Ordinal logistic regression predicting sleep duration with too little to just right touch satisfaction

Predictor	OR	95% confidence interval for OR		SE	*χ* ^2^	*p*
		Lower	Upper			
Gender	0.984	0.909	1.064	0.040	0.165	0.684
Age	1.011	1.008	1.013	0.001	67.815	**<0.001**
End date	0.999	0.997	1.001	0.001	1.781	0.182
Loneliness	1.020	1.016	1.024	0.002	93.552	**<0.001**
Childhood bed routine	0.981	0.957	1.005	0.013	2.351	0.125
Touch recency	0.947	0.910	0.985	0.020	7.306	**0.007**
Touch satisfaction – too little to just right	0.967	0.920	1.017	0.026	1.695	0.193
Attachment avoidance	1.010	1.005	1.016	0.003	15.445	**<0.001**
Attachment anxiety	1.011	1.007	1.016	0.002	24.087	**<0.001**
Touch recency × Attachment avoidance	1.000	0.996	1.005	0.002	0.001	0.979
Touch recency × Attachment anxiety	1.000	0.995	1.004	0.002	0.053	0.818
Touch satisfaction × Attachment avoidance	1.000	0.994	1.006	0.003	0.000	0.986
Touch satisfaction × Attachment anxiety	1.000	0.994	1.005	0.003	0.016	0.899

*Note*: Higher scores indicate shorter sleep duration. For gender, women are the reference category. *N* = 11,624, hence bold fonts significance set at *p* < 0.01.

**TABLE 7:** Ordinal logistic regression predicting WASO number with too little to just right touch satisfaction

Predictor	OR	95% confidence interval for OR		SE	*χ* ^2^	*p*
		Lower	Upper			
Gender	0.896	0.828	0.970	0.040	7.387	**0.007**
Age	1.030	1.027	1.033	0.001	473.502	**<0.001**
End date	0.996	0.994	0.998	0.001	20.925	**<0.001**
Loneliness	1.011	1.007	1.015	0.002	26.949	**<0.001**
Childhood bed routine	0.999	0.975	1.024	0.013	0.004	0.951
Touch recency	0.923	0.887	0.961	0.020	15.616	**<0.001**
Touch satisfaction – too little to just right	0.999	0.949	1.051	0.026	0.002	0.963
Attachment avoidance	1.003	0.998	1.008	0.003	1.194	0.275
Attachment anxiety	1.020	1.015	1.024	0.002	74.799	**<0.001**
Touch recency × Attachment avoidance	1.007	1.002	1.011	0.002	8.771	**0.003**
Touch recency × Attachment anxiety	0.996	0.992	1.000	0.002	3.115	0.078
Touch satisfaction × Attachment avoidance	0.999	0.992	1.005	0.003	0.197	0.657
Touch satisfaction × Attachment anxiety	1.001	0.995	1.007	0.003	0.084	0.771

*Note*: For gender, women are the reference category. *N* = 11,617, hence bold fonts significance set at *p* < 0.01.

**TABLE 8:** Ordinal logistic regression predicting WASO number with too much to just right touch satisfaction

Predictor	OR	95% confidence interval for OR		SE	*χ* ^2^	*p*
		Lower	Upper			
Gender	0.885	0.780	1.004	0.064	3.625	0.057
Age	1.026	1.023	1.030	0.002	186.485	**<0.001**
End date	0.994	0.992	0.997	0.001	16.618	**<0.001**
Loneliness	1.014	1.008	1.021	0.003	17.999	**<0.001**
Childhood bed routine	0.966	0.931	1.002	0.019	3.411	0.065
Touch recency	0.922	0.863	0.985	0.034	5.798	0.016
Touch satisfaction – too much to just right	0.708	0.585	0.857	0.097	12.607	**<0.001**
Attachment avoidance	1.000	0.992	1.008	0.004	0.002	0.966
Attachment anxiety	1.018	1.011	1.025	0.004	25.647	**<0.001**
Touch recency × Attachment avoidance	1.006	1.000	1.013	0.003	3.449	0.063
Touch recency × Attachment anxiety	0.994	0.987	1.001	0.004	2.534	0.111
Touch satisfaction × Attachment avoidance	0.990	0.971	1.009	0.010	1.108	0.292
Touch satisfaction × Attachment anxiety	0.980	0.961	0.999	0.010	4.114	0.043

*Note*: For gender, women are the reference category. *N* = 5410, hence bold fonts significance set at *p* < 0.01.

**TABLE 9:** Ordinal logistic regression predicting WASO duration with too little to just right touch satisfaction

Predictor	OR	95% confidence interval for OR		SE	*χ* ^2^	*p*
		Lower	Upper			
Gender	0.698	0.645	0.756	0.040	79.359	**<0.001**
Age	1.025	1.023	1.028	0.001	357.935	**<0.001**
End date	0.999	0.997	1.001	0.001	1.971	0.160
Loneliness	1.017	1.013	1.022	0.002	74.088	**<0.001**
Childhood bed routine	0.977	0.954	1.001	0.013	3.404	0.065
Touch recency	0.920	0.885	0.957	0.020	17.407	**<0.001**
Touch satisfaction – too little to just right	1.040	0.989	1.094	0.026	2.389	0.122
Attachment avoidance	1.004	0.998	1.009	0.003	1.851	0.174
Attachment anxiety	1.015	1.010	1.019	0.002	41.644	**<0.001**
Touch recency × Attachment avoidance	0.998	0.994	1.003	0.002	0.523	0.470
Touch recency × Attachment anxiety	0.997	0.993	1.001	0.002	1.937	0.164
Touch satisfaction × Attachment avoidance	1.000	0.994	1.007	0.003	0.007	0.931
Touch satisfaction × Attachment anxiety	1.000	0.994	1.006	0.003	0.003	0.957

*Note*: For gender, women are the reference category. *N* = 11,610, hence bold fonts significance set at *p* < 0.01.

**TABLE 10:** Multinomial regression assessing diurnal preference

Predictor	OR	95% confidence interval for OR		SE	*χ* ^2^	*p*
		Lower	Upper			
Definitely a morning type						
Touch recency	0.910	0.818	1.012	0.054	3.012	0.083
Touch satisfaction – too much to just right	1.202	0.900	1.604	0.147	1.554	0.213
Childhood bed routine	0.941	0.882	1.004	0.033	3.413	0.065
Age	1.021	1.015	1.028	0.003	41.208	**0.000**
Attachment avoidance	1.003	0.990	1.017	0.007	0.235	0.628
Attachment anxiety	0.983	0.971	0.994	0.006	8.948	**0.003**
Loneliness	0.996	0.984	1.007	0.006	0.593	0.441
End date	0.997	0.992	1.002	0.002	1.637	0.201
Gender	0.747	0.599	0.931	0.113	6.731	**0.009**
Rather more a morning type than an evening type						
Touch recency	0.850	0.766	0.943	0.053	9.342	0.002
Touch satisfaction – too much to just right	1.134	0.864	1.488	0.139	0.819	0.366
Childhood bed routine	1.017	0.954	1.083	0.032	0.264	0.607
Age	1.017	1.011	1.024	0.003	30.953	<0.001
Attachment avoidance	1.009	0.996	1.022	0.007	1.723	0.189
Attachment anxiety	1.005	0.994	1.016	0.006	0.784	0.376
Loneliness	0.998	0.987	1.008	0.006	0.204	0.651
End date	1.000	0.995	1.005	0.002	0.001	0.970
Gender	0.877	0.710	1.084	0.108	1.476	0.224
Rather more an evening than a morning type						
Touch recency	0.896	0.805	0.998	0.055	4.021	0.045
Touch satisfaction – too much to just right	1.208	0.911	1.601	0.144	1.724	0.189
Childhood bed routine	1.024	0.958	1.093	0.034	0.487	0.485
Age	1.002	0.996	1.008	0.003	0.464	0.496
Attachment avoidance	1.003	0.989	1.017	0.007	0.202	0.653
Attachment anxiety	0.996	0.985	1.007	0.006	0.470	0.493
Loneliness	1.000	0.989	1.011	0.006	0.000	0.991
End date	1.000	0.995	1.005	0.002	0.003	0.957
Gender	0.925	0.745	1.150	0.111	0.492	0.483

*Note*: The reference category is “Definitely an evening type”. For gender, women are the reference category, *N* = 5391, hence bold fonts significance set at *p* < 0.01.

Touch amount satisfaction was not significantly related to sleep outcomes in the analyses using the too little touch scale. However, experiencing too much touch was related to lower sleep quality and higher number of WASOs (Tables 4, 5, 7 and 8). Note that parameter estimates are reported for all analyses for which the relationship between touch recency or touch amount satisfaction and outcomes were significant. The remaining parameter estimates for all conducted regressions are reported in the supplemental material. Also, sample sizes for analyses using the too little touch scale are larger than analyses using the too much scale. This is because more respondents indicated that they receive not enough touch rather than too much touch. Sample sizes are reported in the table captions for each analysis.

**FIGURE 2:**
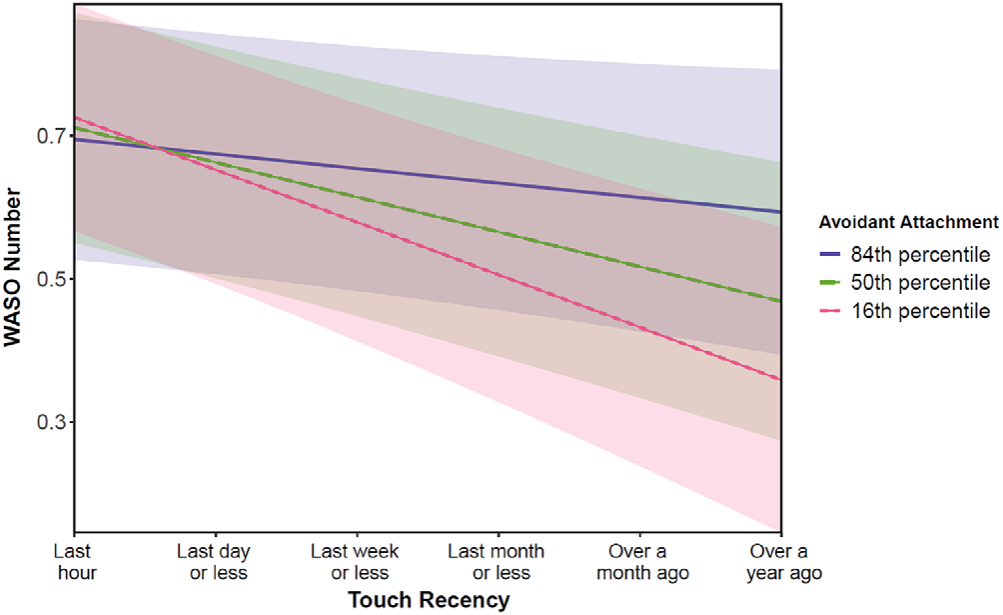
Plot of the interaction effect between touch recency (*x*‐axis) and the low, medium, and high values of avoidant attachment (16th, 50th, 84th percentiles, respectively) on WASO (*y*‐axis)

Furthermore, moderation analyses showed that avoidant attachment significantly moderated the relationship between touch recency and WASO numbers. This relationship, however, was significant only in the analysis using the too little touch scale, and the odds ratio was small (Tables 7 and 8). Overall, individuals reporting more recent touch were more likely to report increased WASO number compared with individuals reporting the last time they received touch further back in time (up to a year ago). The avoidant attachment style further influenced this pattern. Specifically, individuals with low avoidant attachment traits showed a steeper decrease in WASO number when they reported receiving recent touch further back in time than individuals with high avoidance traits (Figure 2).

As reported above, the linear model did not show a good model fit for sleep duration. As both unusually short and long sleep durations have been associated with negative health outcomes (Shankar et al., 2011), the linear model was compared with a quadratic multiple regression model for sleep duration. The quadratic model did not show a significantly better model fit than the linear model, and the only difference in predictors was that touch recency was a significant predictor for sleep duration in the linear but not in the quadratic model with too little touch scale (please see supplemental material).

#### 3.2.2 Hypothesis 3: Childhood bed routine

Participants who agreed more strongly with the statement that they received childhood good‐night hugs and kisses were less likely to categorise themselves as definitely a morning type compared with definitely an evening type.

#### 3.2.3 Hypothesis 4: Gender, age, end date, attachment style and loneliness

Regarding the associations of gender, age, attachment style and loneliness and sleep measurements, our exploratory hypothesis was mostly confirmed. Men indicated better sleep quality than women, however, this difference reached statistical significance only in the too much touch scale analysis. Men indicated shorter sleep latency; in the too much touch scale analysis men indicated significantly fewer WASOs than women. Men moreover reported shorter WASO duration than women. Older people indicated shorter sleep duration, more and longer WASOs, and shorter sleep latency than younger people. Loneliness related to lower sleep quality, shorter sleep duration, higher sleep latency, and longer and more frequent WASOs. Attachment anxiety emerged as a consistent predictor of sleep variables, with greater attachment anxiety relating to poorer sleep quality, shorter sleep duration, greater sleep latency, and higher WASO duration and number of episodes. Higher scores on attachment avoidance were related to shorter sleep duration but not to other sleep variables. Furthermore, later end dates related to better sleep quality and shorter sleep latency in the too little touch scale analysis as well as lower WASO numbers.

Lastly, the multinomial logistic regression to assess the association between touch and diurnal preference showed that men were less likely to classify themselves as a morning type than women. Older people were more likely than younger people to classify themselves as “Definitely a morning type” or “Rather more a morning type than an evening type”. Higher attachment anxiety and later end date related to slightly lower odds of describing oneself as “Definitely a morning type”, though the latter relationship reached significance only in the too little touch scale analysis.

## 4 DISCUSSION

Previous work suggested a relationship between sexual touch before sleep and sleep quality (Dueren et al., 2021). Here, this research was extended by analysing a variety of affective touch experiences (including non‐sexual touch such as hugging) related to sleep outcomes.

### 4.1 Hypothesis 1: How are different types of affective touch judged to influence sleep quality?

Touching a partner during sleep onset was mostly judged to have positive impacts on sleep, followed by negative effects on sleep. These findings might reflect previous research suggesting that there is both a desire for partner touch at sleep onset (Junker et al., 2016), but also the need for private space as touch can impair sleep (Hislop, 2007; Pereira et al., 2017; Sato et al., 2007). The results might relate more broadly to a societal desire to co‐sleep with a partner (National Sleep Foundation, 2005), despite the possible adverse effects of tactile stimuli (Hislop, 2007; Pereira et al., 2017; Sato et al., 2007).

Most respondents ascribed a positive effect on sleep for strokes, hugs, massage, and intimacy. The results regarding intimacy correspond to the perception that sexual intercourse positively influences sleep (Lastella et al., 2019; Pallesen et al., 2020). However, despite this common assumption, it is notable that research on the relationship between those variables has not always reported a positive association between sexual intimacy and sleep (Brissette et al., 1985; Dittami et al., 2007; Seehuus & Pigeon, 2018).

Stroking and hugging were the types of touch for which the least number of participants ascribed impairing sleep effects. Previous evidence suggests that especially slow, caressing stroking has relaxing effects (Morrison, 2016); our findings support the hypothesis that gentle stroking is perceived to aid sleep. Finally, sharing a hug was the type of touch which was least often judged as sleep impairing, in line with emerging evidence suggesting that hugs can have stress‐buffering effects (Morrison, 2016).

### 4.2 Hypothesis 2: How are touch recency and touch amount satisfaction related to sleep variables?

Differences in touch recency were associated with altered levels of subjective sleep variables. Specifically, in the analyses using the too little touch scale, less recent touch was associated with better sleep quality, longer sleep duration, and fewer and shorter WASO, numbers and duration.

The mostly negative associations between touch recency and sleep variables seem to contradict the findings that pre‐sleep touch was judged mainly to impact sleep positively. One explanation for the results could be that participants who indicated a longer time since the most recent touch might live alone. Hence, they might not experience frequent touch and sleep disruptions related to co‐sleeping. Unfortunately, we did not have data to address this possibility. Still, future research may benefit from exploring this notion.

Touch amount satisfaction analyses revealed that too little touch had no significant relationship with sleep outcomes. However, experiencing too much touch was associated with reporting lower sleep quality and a higher number of WASOs. Dissatisfaction with the current amount of affectionate touch has been conceptualised previously in terms of touch starvation (Field, 2014) or, more broadly, as affect deprivation (Floyd, 2014). Interestingly, previous work has found that affect deprivation is linked to impairments in sleep (Floyd, 2016; Hesse et al., 2020). Specifically, it has been argued that humans are adapted to sleep better when they feel secure about their social network (Floyd, 2016). In this context, loneliness and affect deprivation are thought to indicate the lack of functioning social relationships, thereby contributing to heightened insecurity and, consequently, worse sleep quality (Floyd, 2016; Kurina et al., 2011). The current results extend previous findings by showing that a subjective experience of too much touch might also relate to worse sleep outcomes.

It is important to note that we did not find significant relationships between low touch satisfaction and sleep outcomes. One possible reason for this finding could be that experiencing too much touch might have more immediate effects on sleep than the lack of touch. For example, previous qualitative research on the experience of sharing a bed with a romantic partner found that intimate touch before sleep can be perceived as calming, whereas touch during sleep can be perceived as disturbing (Hislop, 2007; Kirkman, 2010). Hislop (2007) writes that partners tend to retreat back to their own side of the bed just prior to sleep onset, because sleep is a solitary activity. The current findings regarding touch amount satisfaction align with such a notion. Nonetheless, it is unclear why experiencing too little touch was not significantly related to sleep outcomes in the current study.

### 4.3 Hypothesis 3: How is childhood bed routine related to sleep variables?

Stronger agreement with the statement that one experienced frequent childhood good night hugs and kisses was associated with a lower odds of classifying oneself as definitely a morning type compared with definitely an evening type in the too little touch scale analysis. Also, a longer time since the last recent touch was associated with lower odds of classifying oneself as rather more a morning type than an evening type in the too much touch scale analysis. However, interpretation of these findings must be cautious as the accuracy of retrospectively reported childhood routines is unclear (Hardt & Rutter, 2004). Furthermore, since other associations between childhood bed routine and sleep outcomes or diurnal preference were not significant, these findings should be considered preliminary.

### 4.4 Hypothesis 4: How are gender, age, attachment style, loneliness and end date related to sleep variables?

In accordance with previous research, women reported slightly worse sleep quality, longer sleep latency and more and longer WASOs than men (Sa et al., 2020). The gender differences in sleep quality and WASO number reached significance only in the too much touch scale analyses. Furthermore, women reported more positive effects of no partner touch during sleep onset than men and vice versa. These results align with previous findings on gender differences in co‐sleeping (Dittami et al., 2007; Kirkman, 2010; Troxel, 2010), suggesting that women are more disturbed by partnered sleep than men.

Men were more likely to ascribe positive effects of intimacy on sleep than women, who were more likely to ascribe negative or neutral effects of intimacy on sleep than men. These findings are in line with previous research suggesting that especially men perceive sex as sleep‐enhancing (Kirkman, 2010; Lastella et al., 2019; Pallesen et al., 2020).

Regarding the relevance of age, in line with previous evidence, older people reported shorter sleep duration and more frequent and longer WASOs (Skeldon et al., 2016).

Furthermore, loneliness has been argued to indicate the lack of functioning social relationships, facilitating feelings of insecurity and worse sleep quality (Floyd, 2016; Kurina et al., 2011). The current findings regarding loneliness align with this hypothesis, as participants who scored higher on the UCLA‐LSR generally indicated lower sleep quality.

Finally, a later end date was associated with greater sleep quality, and reduced sleep latency in the too little touch scale analyses. Furthermore, a later end date was associated with lower WASO numbers. As data were collected between January and the end of March, the overall positive association between a later end date and sleep quality may reflect an overall increase in the ability to self‐regulate sleep patterns, e.g., by lifestyle changes such as working from home following the onset of the first COVID‐19 related lockdown in the UK (Madrid‐Valero et al., 2021).

### 4.5 Limitations and future research

The present analyses have some limitations. As highlighted previously, the data were cross‐sectional, the sample was self‐selecting, and the current results require further investigation to determine the directional nature of associations. The onset of the Covid pandemic during data collection might have impacted results, although most of the data were collected before the first lockdown began in the UK. It is also unknown which participants co‐sleep with family members. Hence some of the interpretations surrounding social sleeping patterns remain speculative. An experimental approach to the current research questions would have allowed us to draw causal inferences about the association between social touch and sleep variables. However, due to pandemic‐related restrictions on experimental work, addressing topics surrounding affective touch in an experimental or laboratory‐based setting was exceedingly difficult at the time of data collection. Thus, the current approach allowed us to investigate the topic of social touch and sleep in a large and diverse group of people despite the subsequent unfolding of the Covid‐19 pandemic. One advantage of the current dataset is the number of interpersonal variables that could be accounted for due to the large sample size. Nevertheless, large‐scale data collection of this kind comes with logistical and methodological costs. Due to time constraints within the wider Touch Test survey, some touch measurements and sleep outcomes investigated in our analyses were assessed via single‐item measures. Single‐item measures have previously been criticised because of a concern that they might not capture complex psychological variables of interest fully (Loo, 2002). However, a meta‐analysis of widely used single‐item measurements reported adequate reliability for single‐item scales that measured homogeneous, unidimensional constructs (Postmes et al., 2013). The constructs measured by single items in the present study mainly represent such unidimensional variables. Exceptions are childhood bed routine, an item that was initially part of the childhood touch subscale of the TEAQ (Trotter et al., 2018), and diurnal preference, which is an item of the MEQ (Horne & Oestberg, 1976). Our assessment of childhood bed routine and diurnal preference was novel and designed to gain insights into questions that have not been addressed before. Therefore, we believe that the use of single items to address these constructs are adequate in the present context. Lastly, it is important to note that many of the measured associations showed small effect sizes. Nevertheless, the present study provides a novel insight into the domain; an interesting future research direction will be to examine whether and how touch interventions may impact sleep quality.

To conclude, the findings reported here shed light on an under‐researched but highly prevalent socially mediated sleep pattern: the impact of touch on sleep. They show a mixed pattern of results highlighting the importance of demographic and psychological individual differences, and type of tactile experience, on sleep outcomes. Several of these relationships warrant future investigation to help to better characterise relationships between touch and sleep.

## AUTHOR CONTRIBUTIONS

The Touch Test was designed by Claudia Hammond, Michael J. Banissy, and Natalie C. Bowling. Study conceptualisation was led by Anna Lena Dueren, Michael J. Banissy, and Alice M. Gregory. Data analysis was conducted by Anna Lena Dueren. All authors contributed to the manuscript.

## CONFLICT OF INTEREST

AMG is an advisor for a project originally partially sponsored by Johnson's Baby. She has written two books (Nodding Off, Bloomsbury Sigma, 2018; The Sleepy Pebble, Flying Eye Books, 2019) and is working on a further project with Lawrence King Publishing. She is a regular contributor to BBC Focus magazine and has contributed to numerous other outlets (such as The Conversation and The Guardian). She has been interviewed by magazines and commercial websites. She has provided a paid talk for business and is occasionally sent trial products from commercial companies (e.g., blue light blocking glasses). She has received grant funding for her research from several bodies. CH give talks for commercial organisations, but not related to the topic of touch and sleep. She is the author of The Art of Rest, published by a commercial publisher. Over the past 3 years MJB has served as a paid consultant to EVE Sleep and provided talks for commercial organisations from a range of sectors. He has contributed to several media outlets worldwide, including work on The Touch Test (a science and broadcast collaboration with BBC Radio 4 funded via the Wellcome Collection). He has also received grant funding from several bodies.

## Supporting information


Appendix S1:

## Data Availability

The data that support the findings of this study are openly available at https://osf.io/ycvbq/?view_only=e1df1439c84a4a93a64e8e712a4e7d63
