# Supplementary material for: Perspectives on interpersonal touch are related to subjective sleep quality
Source: J Sleep Res. 2022 Nov 9;32(3):e13766. doi: 10.1111/jsr.13766 (PMC10909536; doi:10.1111/jsr.13766)
Supplement: Supplementary file 1 — Appendix S1: [file JSR-32-e13766-s001.zip › JSR_13766_supplement_main.docx]

| Table S1: Ordinal logistic regression predicting sleep duration with too much to just right touch satisfaction. For gender, women are the reference category. N = 5413. |  |  |  |  |  |  |
| --- | --- | --- | --- | --- | --- | --- |
|  |  | **95% Confidence Interval for OR** | |  |  |  |
| **Predictor** | **OR** | **Lower** | **Upper** | **SE** | ***χ*^2^** | ***p*** |
| Gender | .974 | .860 | 1.104 | .064 | .168 | .682 |
| Age | 1.008 | 1.004 | 1.012 | .002 | 18.441 | **<.001** |
| End date | .998 | .995 | 1.001 | .001 | 2.133 | .144 |
| Loneliness | 1.017 | 1.010 | 1.023 | .003 | 25.861 | **<.001** |
| Childhood bed routine | .962 | .927 | .998 | .019 | 4.332 | .037 |
| Touch recency | .989 | .927 | 1.056 | .033 | .101 | .751 |
| Touch satisfaction - too much to just right | .852 | .708 | 1.025 | .094 | 2.897 | .089 |
| Attachment avoidance | 1.013 | 1.005 | 1.021 | .004 | 9.889 | **.002** |
| Attachment anxiety | 1.011 | 1.004 | 1.018 | .003 | 9.870 | **.002** |
| Touch recency x Attachment avoidance | 1.001 | .995 | 1.008 | .003 | .136 | .712 |
| Touch recency x Attachment anxiety | 1.003 | .996 | 1.010 | .004 | .825 | .364 |
| Touch satisfaction x Attachment avoidance | .995 | .977 | 1.015 | .010 | .229 | .632 |
| Touch satisfaction x Attachment anxiety | .985 | .966 | 1.005 | .010 | 2.088 | .148 |

| Table S2: Ordinal logistic regression predicting sleep latency with too little to just right touch satisfaction. For gender, women are the reference category. N = 11617. |  |  |  |  |  |  |
| --- | --- | --- | --- | --- | --- | --- |
|  |  | **95% Confidence Interval for OR** | |  |  |  |
| **Predictor** | **OR** | **Lower** | **Upper** | **SE** | ***χ*^2^** | ***p*** |
| Gender | .630 | .579 | .686 | .043 | 115.477 | **<.001** |
| Age | .995 | .993 | .998 | .001 | 11.571 | **.001** |
| End date | .996 | .995 | .998 | .001 | 13.698 | **<.001** |
| Loneliness | 1.015 | 1.011 | 1.019 | .002 | 48.365 | **<.001** |
| Childhood bed routine | .969 | .944 | .993 | .013 | 6.067 | .014 |
| Touch recency | 1.051 | 1.009 | 1.095 | .021 | 5.794 | .016 |
| Touch satisfaction - too little to just right | 1.043 | .990 | 1.099 | .027 | 2.530 | .112 |
| Attachment avoidance | 1.002 | .997 | 1.008 | .003 | .679 | .410 |
| Attachment anxiety | 1.020 | 1.016 | 1.025 | .002 | 74.220 | **<.001** |
| Touch recency x Attachment avoidance | .999 | .995 | 1.004 | .002 | .061 | .805 |
| Touch recency x Attachment anxiety | 1.000 | .995 | 1.004 | .002 | .009 | .924 |
| Touch satisfaction x Attachment avoidance | 1.005 | .998 | 1.011 | .003 | 2.046 | .153 |
| Touch satisfaction x Attachment anxiety | 1.004 | .998 | 1.010 | .003 | 1.712 | .191 |

| Table S3: Ordinal logistic regression predicting sleep latency with too much to just right touch satisfaction. For gender, women are the reference category. N = 5409. |  |  |  |  |  |  |
| --- | --- | --- | --- | --- | --- | --- |
|  |  | **95% Confidence Interval for OR** | |  |  |  |
| **Predictor** | **OR** | **Lower** | **Upper** | **SE** | ***χ*^2^** | ***p*** |
| Gender | .656 | .573 | .750 | .069 | 37.637 | **<.001** |
| Age | .994 | .990 | .997 | .002 | 11.634 | **.001** |
| End date | .998 | .995 | 1.001 | .001 | 2.170 | .141 |
| Loneliness | 1.016 | 1.010 | 1.023 | .003 | 22.794 | **<.001** |
| Childhood bed routine | .974 | .937 | 1.012 | .020 | 1.873 | .171 |
| Touch recency | 1.067 | .998 | 1.141 | .034 | 3.623 | .057 |
| Touch satisfaction - too much to just right | .960 | .792 | 1.164 | .098 | .174 | .677 |
| Attachment avoidance | 1.007 | .999 | 1.015 | .004 | 2.778 | .096 |
| Attachment anxiety | 1.024 | 1.017 | 1.031 | .004 | 44.209 | **<.001** |
| Touch recency x Attachment avoidance | 1.001 | .994 | 1.008 | .003 | .070 | .792 |
| Touch recency x Attachment anxiety | 1.003 | .996 | 1.010 | .004 | .642 | .423 |
| Touch satisfaction x Attachment avoidance | .975 | .956 | .995 | .010 | 6.211 | .013 |
| Touch satisfaction x Attachment anxiety | .999 | .978 | 1.019 | .010 | .016 | .900 |

| Table S4: Ordinal logistic regression predicting WASO duration with too much to just right touch satisfaction. For gender, women are the reference category. N = 5407. |  |  |  |  |  |  |
| --- | --- | --- | --- | --- | --- | --- |
|  |  | **95% Confidence Interval for OR** | |  |  |  |
| **Predictor** | **OR** | **Lower** | **Upper** | **SE** | ***χ*^2^** | ***p*** |
| Gender | .700 | .618 | .794 | .064 | 30.813 | **<.001** |
| Age | 1.026 | 1.022 | 1.030 | .002 | 182.717 | **<.001** |
| End date | .998 | .995 | 1.001 | .001 | 1.802 | .179 |
| Loneliness | 1.021 | 1.015 | 1.028 | .003 | 41.479 | **<.001** |
| Childhood bed routine | .965 | .930 | 1.001 | .019 | 3.649 | .056 |
| Touch recency | .918 | .860 | .980 | .033 | 6.598 | .010 |
| Touch satisfaction - too much to just right | .924 | .767 | 1.114 | .095 | .683 | .409 |
| Attachment avoidance | 1.003 | .995 | 1.011 | .004 | .480 | .489 |
| Attachment anxiety | 1.011 | 1.004 | 1.018 | .003 | 10.191 | **.001** |
| Touch recency x Attachment avoidance | .992 | .986 | .999 | .003 | 5.388 | .020 |
| Touch recency x Attachment anxiety | .997 | .990 | 1.004 | .004 | .557 | .456 |
| Touch satisfaction x Attachment avoidance | .996 | .978 | 1.015 | .009 | .157 | .692 |
| Touch satisfaction x Attachment anxiety | 1.003 | .983 | 1.023 | .010 | .079 | .779 |

| Table S5: Multinomial regression assessing diurnal preference with too little to just right touch amount. The reference category is "Definitely an evening type". For gender, women are the reference category, *N* = 11589. | | | | | | |
| --- | --- | --- | --- | --- | --- | --- |
|  | **Definitely a morning type** | | |  |  |  |
|  |  | **95% Confidence Interval for OR** | |  |  |  |
| **Predictor** | **OR** | **Lower** | **Upper** | **SE** | ***χ*^2^** | ***p*** |
| Touch recency | .944 | .882 | 1.009 | .034 | 2.857 | .091 |
| Touch satisfaction - too little to just right | 1.024 | .937 | 1.118 | .045 | .269 | .604 |
| Childhood bed routine | .934 | .893 | .975 | .022 | 9.444 | .002 |
| Age | 1.022 | 1.017 | 1.027 | .002 | 86.820 | .000 |
| Attachment avoidance | 1.006 | .997 | 1.015 | .005 | 1.756 | .185 |
| Attachment anxiety | .984 | .976 | .992 | .004 | 16.412 | .000 |
| Loneliness | 1.000 | .993 | 1.007 | .004 | .001 | .978 |
| End date | .994 | .991 | .998 | .002 | 11.577 | .001 |
| Gender | .747 | .651 | .859 | .071 | 16.936 | .000 |
|  | **Rather more a morning type than an evening type** | | | | |  |
|  |  | **95% Confidence Interval for OR** | |  |  |  |
|  | **OR** | **Lower** | **Upper** | **SE** | ***χ*^2^** | ***p*** |
| Touch recency | .948 | .888 | 1.012 | .033 | 2.578 | .108 |
| Touch satisfaction - too little to just right | 1.057 | .970 | 1.151 | .044 | 1.585 | .208 |
| Childhood bed routine | 1.003 | .961 | 1.047 | .022 | .016 | .898 |
| Age | 1.016 | 1.012 | 1.020 | .002 | 51.997 | .000 |
| Attachment avoidance | 1.002 | .994 | 1.011 | .005 | .302 | .583 |
| Attachment anxiety | .999 | .991 | 1.006 | .004 | .145 | .703 |
| Loneliness | 1.003 | .996 | 1.010 | .003 | .607 | .436 |
| End date | .997 | .994 | 1.000 | .002 | 3.612 | .057 |
| Gender | .808 | .707 | .923 | .068 | 9.815 | .002 |
|  | **Rather more an evening type than a morning type** | | | | |  |
|  |  | **95% Confidence Interval for OR** | |  |  |  |
|  | **OR** | **Lower** | **Upper** | **SE** | ***χ*^2^** | ***p*** |
| Touch recency | .957 | .895 | 1.023 | .034 | 1.690 | .194 |
| Touch satisfaction - too little to just right | 1.003 | .919 | 1.096 | .045 | .006 | .939 |
| Childhood bed routine | .989 | .946 | 1.033 | .023 | .261 | .609 |
| Age | 1.004 | .999 | 1.008 | .002 | 2.482 | .115 |
| Attachment avoidance | .997 | .988 | 1.006 | .005 | .332 | .564 |
| Attachment anxiety | .997 | .989 | 1.005 | .004 | .662 | .416 |
| Loneliness | 1.004 | .997 | 1.011 | .004 | 1.360 | .244 |
| End date | .998 | .995 | 1.001 | .002 | 1.226 | .268 |
| Gender | .857 | .748 | .983 | .070 | 4.893 | .027 |

| Table S6: Regression models to predict sleep duration with linear predictors in model 1 and squared predictors in model 2 with too little touch scale. Moderation terms have been left out for ease of interpretation. | | | | | |
| --- | --- | --- | --- | --- | --- |
| **Predictor** | **B** | **SE(B)** | **Beta** | **t** | ***p*** |
| **Model 1** |  |  |  |  |  |
| Touch recency | -.030 | .011 | -.029 | -2.789 | **.005** |
| Touch satisfaction - too little to just right | -.014 | .014 | -.010 | -1.002 | .317 |
| Childhood Bed routine | -.013 | .007 | -.018 | -1.922 | .055 |
| Gender | .013 | .022 | .005 | .568 | .570 |
| Age | .006 | .001 | .077 | 8.095 | **<.001** |
| Attachment Avoidance | .005 | .001 | .039 | 3.823 | **<.001** |
| Attachment Anxiety | .006 | .001 | .049 | 4.890 | **<.001** |
| Loneliness | .011 | .001 | .112 | 9.585 | **<.001** |
| End Date | -.001 | .001 | -.011 | -1.224 | .221 |
| **Model 2** |  |  |  |  |  |
| Touch recency | -.027 | .035 | -.027 | -.788 | .431 |
| Touch satisfaction - too little to just right | -.017 | .016 | -.013 | -1.121 | .263 |
| Childhood Bed routine | -.019 | .039 | -.026 | -.484 | .628 |
| Gender | .012 | .022 | .005 | .556 | .578 |
| Age | .006 | .001 | .077 | 8.095 | **<.001** |
| Attachment Avoidance | .005 | .001 | .039 | 3.759 | **<.001** |
| Attachment Anxiety | .006 | .001 | .049 | 4.876 | **<.001** |
| Loneliness | .011 | .001 | .112 | 9.551 | **<.001** |
| End Date | -.001 | .001 | -.011 | -1.224 | .221 |
| Touch recency squared | .000 | .006 | -.002 | -.067 | .946 |
| Touch satisfaction - too little to just right squared | -.011 | .020 | -.006 | -.539 | .590 |
| Childhood Bed routine squared | .001 | .006 | .008 | .147 | .883 |

| Table S7: Model summaries of linear and quadratic model to predict sleep duration with too little touch scale. | | | | | | | | | |  | |
| --- | --- | --- | --- | --- | --- | --- | --- | --- | --- | --- | --- |
|  |  |  |  |  | **Change statistics** | | | | | |  |
| **Model** | **R** | **R²** | **Adj R²** | **SE(B)** | **ΔR^2^** | **ΔF** | **df1** | **df2** | **Δ*p*** | |  |
| Linear model | .171 | .029 | .028 | 1.005 | .029 | 38.863 | 9.000 | 11614.000 | **<.001** | |  |
| Quadratic model | .171 | .029 | .028 | 1.005 | .000 | .106 | 3.000 | 11611.000 | .956 | |  |

| Table S8: Regression models to predict sleep duration with linear predictors in model 1 and squared predictors in model 2 with too much touch scale. Moderation terms have been left out for ease of interpretation. | | | | | |
| --- | --- | --- | --- | --- | --- |
| **Predictor** | **B** | **SE(B)** | **Beta** | **t** | ***p*** |
| **Model 1** |  |  |  |  |  |
| Touch recency | -.009 | .017 | -.008 | -.521 | .602 |
| Touch satisfaction - too much to just right | -.090 | .047 | -.026 | -1.906 | .057 |
| Childhood Bed routine | -.022 | .010 | -.030 | -2.145 | .032 |
| Gender | .016 | .035 | .006 | .470 | .638 |
| Age | .004 | .001 | .057 | 4.092 | **<.001** |
| Attachment Avoidance | .006 | .002 | .046 | 2.946 | **.003** |
| Attachment Anxiety | .005 | .002 | .040 | 2.796 | **.005** |
| Loneliness | .009 | .002 | .083 | 5.135 | **<.001** |
| End Date | -.001 | .001 | -.017 | -1.295 | .195 |
| **Model 2** |  |  |  |  |  |
| Touch recency | -.052 | .051 | -.044 | -1.017 | .309 |
| Touch satisfaction - too much to just right | -.032 | .125 | -.009 | -.259 | .796 |
| Childhood Bed routine | -.079 | .059 | -.109 | -1.352 | .176 |
| Gender | .015 | .035 | .006 | .430 | .667 |
| Age | .004 | .001 | .058 | 4.095 | **<.001** |
| Attachment Avoidance | .006 | .002 | .046 | 2.992 | **.003** |
| Attachment Anxiety | .005 | .002 | .041 | 2.843 | **.004** |
| Loneliness | .009 | .002 | .083 | 5.094 | **<.001** |
| End Date | -.001 | .001 | -.017 | -1.288 | .198 |
| Touch recency squared | .009 | .010 | .039 | .891 | .373 |
| Touch satisfaction - too much to just right squared | .043 | .092 | .017 | .468 | .639 |
| Childhood Bed routine squared | .009 | .009 | .080 | .990 | .322 |

| Table S9: Model summaries of linear and quadratic model to predict sleep duration with too little touch scale. | | | | | | | | |  |
| --- | --- | --- | --- | --- | --- | --- | --- | --- | --- |
|  |  |  |  |  | **Change statistics** | | | | |
| **Model** | **R** | **R²** | **Adj R²** | **SE(B)** | **ΔR^2^** | **ΔF** | **df1** | **df2** | **Δ*p*** |
| Linear model | .148 | .022 | .020 | .994 | .022 | 13.434 | 9.000 | 5403.000 | **<.001** |
| Quadratic model | .149 | .022 | .020 | .995 | .000 | .720 | 3.000 | 5400.000 | .540 |
