# Supplementary material for: Perspectives on interpersonal touch are related to subjective sleep quality
Source: J Sleep Res. 2022 Nov 9;32(3):e13766. doi: 10.1111/jsr.13766 (PMC10909536; doi:10.1111/jsr.13766)
Supplement: Supplementary file 1 — Appendix S1: [file JSR-32-e13766-s001.zip › JSR_13766_supplement_sensitivity_analyses.docx]

Supplementary Results: Sensitivity analyses

Hypothesis 1: For participants who had experienced touch recently, statistics for the Chi-square goodness-of-fit tests assessing distribution across judgments were all significant at *p* < .001 with test statistics as followed: Stroke *χ*2(2) = 5126.720, Hug *χ*2(2) = 4993.464, Massage *χ*2(2) = 1736.545, Intimacy *χ*2(2) = 1550.723, No touch at sleep onset *χ*2(2) = 712.108, Touch at sleep onset *χ*2(2) = 861.741.

For participants who had not experienced touch recently, statistics for the Chi-square goodness-of-fit tests assessing distribution across judgments were also significant at *p* < .001 with test statistics as followed: Stroke *χ*2(2) = 311.125, Hug *χ*2(2) = 245.033, Massage *χ*2(2) = 69.361, Intimacy *χ*2(2) = 115.629, No touch at sleep onset *χ*2(2) = 52.627, Touch at sleep onset *χ*2(2) = 96.357.

Table S10: Participants who have experienced touch recently. Observed proportions in percent for pairwise comparisons with binomial tests. Significance levels are Bonferroni corrected. Note that the answers given for the third category are not considered here to facilitate pairwise comparison, therefore observed proportions add up to 100%.

| **Comparison** |  |  | **Stroke** | **Hug** | **Massage** | **Intimacy** | **Not touching partner at sleep onset** | **Touching partner at sleep onset** |
| --- | --- | --- | --- | --- | --- | --- | --- | --- |
| Negative vs neutral | Observed proportion | negative | 27% | 8% | 49% | 53% | 34% | 66% |
|  |  | neutral | 73% | 92% | 51% | 47% | 66% | 34% |
|  | *p* |  | **<.001** | **<.001** | 1.518 | **<.001** | **<.001** | **<.001** |
| Neutral vs positive | Observed proportion | neutral | 27% | 37% | 30% | 30% | 52% | 32% |
|  |  | positive | 73% | 63% | 70% | 70% | 48% | 68% |
|  | *p* |  | **<.001** | **<.001** | **<.001** | **<.001** | **.003** | **<.001** |
| Negative vs positive | Observed proportion | negative | 12% | 5% | 30% | 33% | 35% | 48% |
|  |  | positive | 88% | 95% | 70% | 67% | 65% | 52% |
|  | *p* |  | **<.001** | **<.001** | **<.001** | **<.001** | **<.001** | **<.001** |
| N per analysis | | | 9618 | 9740 | 9419 | 9468 | 9803 | 9916 |

Table S11: Participants who have not experienced touch recently. Observed proportions in percent for pairwise comparisons with binomial tests. Significance levels are Bonferroni corrected. Note that the answers given for the third category are not considered here to facilitate pairwise comparison, therefore observed proportions add up to 100%.

| **Comparison** |  |  | **Stroke** | **Hug** | **Massage** | **Intimacy** | **Not touching partner at sleep onset** | **Touching partner at sleep onset** |
| --- | --- | --- | --- | --- | --- | --- | --- | --- |
| Negative vs neutral | Observed proportion | negative | 35% | 17% | 61% | 64% | 35% | 71% |
|  |  | neutral | 65% | 83% | 39% | 36% | 65% | 29% |
|  | *p* |  | **<.001** | **<.001** | **<.001** | **<.001** | **<.001** | **<.001** |
| Neutral vs positive | Observed proportion | neutral | 29% | 44% | 32% | 27% | 54% | 31% |
|  |  | positive | 71% | 56% | 68% | 73% | 46% | 69% |
|  | *p* |  | **<.001** | **.006** | **<.001** | **<.001** | .124 | **<.001** |
| Negative vs positive | Observed proportion | negative | 18% | 14% | 42% | 40% | 38% | 49% |
|  |  | positive | 82% | 86% | 58% | 60% | 62% | 51% |
|  | *p* |  | **<.001** | **<.001** | **<.001** | **<.001** | **<.001** | 1.356 |
| N per analysis | | | 814 | 811 | 789 | 793 | 851 | 858 |

Table S12: Participants who have experienced touch recently. Chi-square test of independence comparing touch judgments across men and women. Adjusted residuals are considered to indicate a significant difference when they are < -3 or >3.

| \|  \|  \| **Stroke** \| \| **Hug** \| \| **Massage** \| \| **Intimacy** \| \| \| --- \| --- \| --- \| --- \| --- \| --- \| --- \| --- \| --- \| --- \| \| **Judgment** \|  \| **Men** \| **Women** \| **Men** \| **Women** \| **Men** \| **Women** \| **Men** \| **Women** \| \| Negative effect on sleep \| Percent responses \| 1.6% \| 7.5% \| 0.6% \| 2.4% \| 3.8% \| 19.2% \| 3.8% \| 21.5% \| \|  \| Adjusted Residual \| **-4.2** \| **4.2** \| -0.7 \| 0.7 \| **-8.0** \| **8.0** \| **-11.0** \| **11.0** \| \| No effect on sleep \| Percent responses \| 5.2% \| 19.0% \| 7.9% \| 27.6% \| 5.8% \| 17.7% \| 4.0% \| 18.4% \| \|  \| Adjusted Residual \| -2.0 \| 2.0 \| -1.0 \| 1.0 \| 2.4 \| -2.4 \| **-6.7** \| **6.7** \| \| Positive effect on sleep \| Percent responses \| 16.2% \| 49.4% \| 14.3% \| 47.1% \| 13.3% \| 40.3% \| 15.5% \| 36.9% \| \|  \| Adjusted Residual \| **4.4** \| **-4.4** \| 1.3 \| -1.3 \| **4.7** \| **-4.7** \| **15.2** \| **-15.2** \| \|  \| Chi-square test of independence \| χ^2^(2) = 25.316, *p* < .001 \| \| χ^2^(2) = 1.811, *p =* .404 \| \| χ^2^(2) = 64.272, *p* < .001 \| \| χ^2^(2) = 236.376, *p* < .001 \| \| \|  \|  \| **No touch at sleep onset** \| \| **Touch at sleep onset** \| \|  \|  \|  \|  \| \|  \|  \| **Men** \| **Women** \| **Men** \| **Women** \|  \|  \|  \|  \| \| Negative effect on sleep \| Percent responses \| 5.0% \| 15.7% \| 7.3% \| 31.0% \|  \|  \|  \|  \| \|  \| Adjusted Residual \| 1.7 \| -1.7 \| **-7.0** \| **7.0** \|  \|  \|  \|  \| \| No effect on sleep \| Percent responses \| 11.0% \| 30.1% \| 4.8% \| 14.9% \|  \|  \|  \|  \| \|  \| Adjusted Residual \| **7.6** \| **-7.6** \| 1.7 \| -1.7 \|  \|  \|  \|  \| \| Positive effect on sleep \| Percent responses \| 6.9% \| 31.3% \| 10.7% \| 31.4% \|  \|  \|  \|  \| \|  \| Adjusted Residual \| **-9.1** \| **9.1** \| **5.5** \| **-5.5** \|  \|  \|  \|  \| \|  \| Chi-square test of independence \| χ^2^(2) = 87.343, *p* < .001 \| \| χ^2^(2) = 50.022, *p* < .001 \| \|  \|  \|  \|  \| |
| --- | --- | --- | --- | --- | --- | --- | --- | --- | --- | --- | --- | --- | --- | --- | --- | --- | --- | --- | --- | --- | --- | --- | --- | --- | --- | --- | --- | --- | --- | --- | --- | --- | --- | --- | --- | --- | --- | --- | --- | --- | --- | --- | --- | --- | --- | --- | --- | --- | --- | --- | --- | --- | --- | --- | --- | --- | --- | --- | --- | --- | --- | --- | --- | --- | --- | --- | --- | --- | --- | --- | --- | --- | --- | --- | --- | --- | --- | --- | --- | --- | --- | --- | --- | --- | --- | --- | --- | --- | --- | --- | --- | --- | --- | --- | --- | --- | --- | --- | --- | --- | --- | --- | --- | --- | --- | --- | --- | --- | --- | --- | --- | --- | --- | --- | --- | --- | --- | --- | --- | --- | --- | --- | --- | --- | --- | --- | --- | --- | --- | --- | --- | --- | --- | --- | --- | --- | --- | --- | --- | --- | --- | --- | --- | --- | --- | --- | --- | --- | --- | --- | --- | --- | --- | --- | --- | --- | --- | --- | --- | --- | --- | --- | --- | --- | --- | --- | --- | --- | --- | --- | --- | --- | --- | --- | --- | --- | --- | --- | --- | --- |

Table S13: Participants who have not experienced touch recently. Chi-square test of independence comparing touch judgments across men and women. Adjusted residuals are considered to indicate a significant difference when they are < -3 or >3.

| \|  \|  \| **Stroke** \| \| **Hug** \| \| **Massage** \| \| **Intimacy** \| \| \| --- \| --- \| --- \| --- \| --- \| --- \| --- \| --- \| --- \| --- \| \| **Judgment** \|  \| **Men** \| **Women** \| **Men** \| **Women** \| **Men** \| **Women** \| **Men** \| **Women** \| \| Negative effect on sleep \| Percent responses \| 4.1% \| 9.2% \| 2.2% \| 5.9% \| 10.6% \| 22.6% \| 9.3% \| 23.2% \| \|  \| Adjusted Residual \| -1.9 \| 1.9 \| -2.1 \| 2.1 \| -2.8 \| 2.8 \| **-4.4** \| **4.4** \| \| No effect on sleep \| Percent responses \| 10.1% \| 15.0% \| 17.0% \| 23.7% \| 10.0% \| 11.3% \| 7.2% \| 11.0% \| \|  \| Adjusted Residual \| 0.4 \| -0.4 \| 1.3 \| -1.3 \| 2.4 \| -2.4 \| 0.0 \| 0.0 \| \| Positive effect on sleep \| Percent responses \| 24.8% \| 36.9% \| 19.9% \| 31.3% \| 18.3% \| 27.2% \| 23.1% \| 26.2% \| \|  \| Adjusted Residual \| 1.0 \| -1.0 \| -0.2 \| 0.2 \| 0.6 \| -0.6 \| **4.1** \| **-4.1** \| \|  \| Chi-square test of independence \| χ^2^(2) = 3.685, *p* =.158 \| \| χ^2^(2) = 4.918, *p =* .086 \| \| χ^2^(2) = 10.041, *p* = .007 \| \| χ^2^(2) = 21.340, *p* < .001 \| \| \|  \|  \| **No touch at sleep onset** \| \| **Touch at sleep onset** \| \|  \|  \|  \|  \| \|  \|  \| **Men** \| **Women** \| **Men** \| **Women** \|  \|  \|  \|  \| \| Negative effect on sleep \| Percent responses \| 8.5% \| 13.6% \| 14.2% \| 28.2% \|  \|  \|  \|  \| \|  \| Adjusted Residual \| -0.4 \| 0.4 \| -2.7 \| 2.7 \|  \|  \|  \|  \| \| No effect on sleep \| Percent responses \| 18.1% \| 23.7% \| 8.5% \| 9.1% \|  \|  \|  \|  \| \|  \| Adjusted Residual \| 1.9 \| -1.9 \| 2.6 \| -2.6 \|  \|  \|  \|  \| \| Positive effect on sleep \| Percent responses \| 13.0% \| 23.0% \| 16.1% \| 23.9% \|  \|  \|  \|  \| \|  \| Adjusted Residual \| -1.5 \| 1.5 \| 0.7 \| -0.7 \|  \|  \|  \|  \| \|  \| Chi-square test of independence \| χ^2^(2) = 3.647, *p* =.161 \| \| χ^2^(2) = 10.368, *p* = .006 \| \|  \|  \|  \|  \| |
| --- | --- | --- | --- | --- | --- | --- | --- | --- | --- | --- | --- | --- | --- | --- | --- | --- | --- | --- | --- | --- | --- | --- | --- | --- | --- | --- | --- | --- | --- | --- | --- | --- | --- | --- | --- | --- | --- | --- | --- | --- | --- | --- | --- | --- | --- | --- | --- | --- | --- | --- | --- | --- | --- | --- | --- | --- | --- | --- | --- | --- | --- | --- | --- | --- | --- | --- | --- | --- | --- | --- | --- | --- | --- | --- | --- | --- | --- | --- | --- | --- | --- | --- | --- | --- | --- | --- | --- | --- | --- | --- | --- | --- | --- | --- | --- | --- | --- | --- | --- | --- | --- | --- | --- | --- | --- | --- | --- | --- | --- | --- | --- | --- | --- | --- | --- | --- | --- | --- | --- | --- | --- | --- | --- | --- | --- | --- | --- | --- | --- | --- | --- | --- | --- | --- | --- | --- | --- | --- | --- | --- | --- | --- | --- | --- | --- | --- | --- | --- | --- | --- | --- | --- | --- | --- | --- | --- | --- | --- | --- | --- | --- | --- | --- | --- | --- | --- | --- | --- | --- | --- | --- | --- | --- | --- | --- | --- | --- | --- | --- | --- |

| Table S14: Ordinal logistic regression predicting sleep quality with too little to just right touch satisfaction, including participants who experienced more recent touch. For gender, women are the reference category. N = 10685. | | | | | | |
| --- | --- | --- | --- | --- | --- | --- |
| **Sleep quality** | | | | | | |
|  |  | **95% Confidence Interval for OR** | |  |  |  |
| **Predictor** | **OR** | **Lower** | **Upper** | **SE** | ***χ*^2^** | ***p*** |
| Gender | .903 | .827 | .985 | .045 | 5.233 | .022 |
| Age | 1.001 | .998 | 1.004 | .001 | .519 | .471 |
| End date | .996 | .994 | .998 | .001 | 12.324 | **<.001** |
| Loneliness | 1.033 | 1.028 | 1.038 | .002 | 198.276 | **<.001** |
| Childhood bed routine | .976 | .949 | 1.003 | .014 | 3.164 | .075 |
| Touch recency | .909 | .858 | .962 | .029 | 10.745 | **.001** |
| Touch satisfaction - too little to just right | .942 | .891 | .996 | .028 | 4.429 | .035 |
| Attachment avoidance | 1.003 | .997 | 1.009 | .003 | .972 | .324 |
| Attachment anxiety | 1.025 | 1.019 | 1.030 | .003 | 88.983 | **<.001** |
| Touch recency x Attachment avoidance | .994 | .986 | 1.002 | .004 | 2.344 | .126 |
| Touch recency x Attachment anxiety | 1.005 | .998 | 1.011 | .003 | 1.701 | .192 |
| Touch satisfaction x Attachment avoidance | 1.007 | .999 | 1.014 | .004 | 3.302 | .069 |
| Touch satisfaction x Attachment anxiety | .999 | .993 | 1.006 | .003 | .030 | .863 |

| Table S15: Ordinal logistic regression predicting sleep quality with too much to just right touch satisfaction, including participants who experienced more recent touch. For gender, women are the reference category. N = 5203. | | | | | | |
| --- | --- | --- | --- | --- | --- | --- |
| **Sleep quality** | | | | | | |
|  |  | **95% Confidence Interval for OR** | |  |  |  |
| **Predictor** | **OR** | **Lower** | **Upper** | **SE** | ***χ*^2^** | ***p*** |
| Gender | .832 | .726 | .954 | .070 | 6.925 | **.008** |
| Age | .999 | .995 | 1.002 | .002 | .481 | .488 |
| End date | .997 | .994 | 1.000 | .001 | 3.184 | .074 |
| Loneliness | 1.038 | 1.031 | 1.045 | .004 | 105.830 | **<.001** |
| Childhood bed routine | .977 | .939 | 1.016 | .020 | 1.339 | .247 |
| Touch recency | .963 | .881 | 1.051 | .045 | .717 | .397 |
| Touch satisfaction - too much to just right | .689 | .560 | .846 | .105 | 12.565 | **<.001** |
| Attachment avoidance | 1.004 | .994 | 1.013 | .005 | .598 | .439 |
| Attachment anxiety | 1.024 | 1.016 | 1.033 | .004 | 32.673 | **<.001** |
| Touch recency x Attachment avoidance | .991 | .979 | 1.002 | .006 | 2.488 | .115 |
| Touch recency x Attachment anxiety | 1.007 | .997 | 1.018 | .005 | 1.921 | .166 |
| Touch satisfaction x Attachment avoidance | 1.014 | .991 | 1.036 | .011 | 1.394 | .238 |
| Touch satisfaction x Attachment anxiety | .976 | .954 | .998 | .012 | 4.472 | .034 |

| Table S16: Ordinal logistic regression predicting sleep duration with too little to just right touch satisfaction, including participants who experienced more recent touch. For gender, women are the reference category. N = 10684. | | | | | | |
| --- | --- | --- | --- | --- | --- | --- |
| **Sleep duration** | | | | | | |
|  |  | **95% Confidence Interval for OR** | |  |  |  |
| **Predictor** | **OR** | **Lower** | **Upper** | **SE** | ***χ*^2^** | ***p*** |
| Gender | .989 | .910 | 1.075 | .043 | .069 | .793 |
| Age | 1.012 | 1.009 | 1.015 | .001 | 78.130 | **<.001** |
| End date | .999 | .997 | 1.001 | .001 | 1.986 | .159 |
| Loneliness | 1.020 | 1.016 | 1.024 | .002 | 82.666 | **<.001** |
| Childhood bed routine | .983 | .958 | 1.009 | .013 | 1.731 | .188 |
| Touch recency | .951 | .900 | 1.004 | .028 | 3.294 | .070 |
| Touch satisfaction - too little to just right | .964 | .914 | 1.016 | .027 | 1.872 | .171 |
| Attachment avoidance | 1.009 | 1.003 | 1.015 | .003 | 9.484 | **.002** |
| Attachment anxiety | 1.013 | 1.008 | 1.018 | .002 | 28.737 | **<.001** |
| Touch recency x Attachment avoidance | .995 | .987 | 1.002 | .004 | 1.857 | .173 |
| Touch recency x Attachment anxiety | 1.005 | .998 | 1.012 | .003 | 2.260 | .133 |
| Touch satisfaction x Attachment avoidance | 1.000 | .993 | 1.007 | .004 | .001 | .971 |
| Touch satisfaction x Attachment anxiety | 1.000 | .994 | 1.006 | .003 | .002 | .965 |

| Table S17: Ordinal logistic regression predicting sleep duration with too much to just right touch satisfaction, including participants who experienced more recent touch. For gender, women are the reference category. N = 5202. | | | | | | |
| --- | --- | --- | --- | --- | --- | --- |
| **Sleep duration** | | | | | | |
|  |  | **95% Confidence Interval for OR** | |  |  |  |
| **Predictor** | **OR** | **Lower** | **Upper** | **SE** | ***χ*^2^** | ***p*** |
| Gender | .972 | .855 | 1.105 | .066 | .186 | .666 |
| Age | 1.009 | 1.005 | 1.013 | .002 | 21.898 | **<.001** |
| End date | .998 | .995 | 1.001 | .001 | 2.392 | .122 |
| Loneliness | 1.017 | 1.010 | 1.024 | .003 | 23.948 | **<.001** |
| Childhood bed routine | .966 | .930 | 1.003 | .019 | 3.312 | .069 |
| Touch recency | .972 | .893 | 1.058 | .043 | .430 | .512 |
| Touch satisfaction - too much to just right | .844 | .697 | 1.022 | .098 | 3.035 | .081 |
| Attachment avoidance | 1.012 | 1.003 | 1.021 | .005 | 7.096 | **.008** |
| Attachment anxiety | 1.013 | 1.005 | 1.021 | .004 | 9.635 | **.002** |
| Touch recency x Attachment avoidance | .997 | .986 | 1.008 | .006 | .265 | .607 |
| Touch recency x Attachment anxiety | 1.006 | .996 | 1.016 | .005 | 1.435 | .231 |
| Touch satisfaction x Attachment avoidance | 1.009 | .989 | 1.030 | .010 | .791 | .374 |
| Touch satisfaction x Attachment anxiety | .985 | .964 | 1.006 | .011 | 1.974 | .160 |

| Table S18: Ordinal logistic regression predicting sleep latency with too little to just right touch satisfaction, including participants who experienced more recent touch. For gender, women are the reference category. N = 10678. | | | | | | |
| --- | --- | --- | --- | --- | --- | --- |
| **Sleep latency** | | | | | | |
|  |  | **95% Confidence Interval for OR** | |  |  |  |
| **Predictor** | **OR** | **Lower** | **Upper** | **SE** | ***χ*^2^** | ***p*** |
| Gender | .644 | .589 | .704 | .045 | 93.424 | **<.001** |
| Age | .996 | .993 | .998 | .001 | 10.091 | **.001** |
| End date | .996 | .994 | .998 | .001 | 12.719 | **<.001** |
| Loneliness | 1.014 | 1.010 | 1.019 | .002 | 40.042 | **<.001** |
| Childhood bed routine | .964 | .939 | .990 | .014 | 7.214 | **.007** |
| Touch recency | 1.033 | .976 | 1.093 | .029 | 1.239 | .266 |
| Touch satisfaction - too little to just right | 1.035 | .979 | 1.093 | .028 | 1.461 | .227 |
| Attachment avoidance | 1.001 | .996 | 1.007 | .003 | .215 | .643 |
| Attachment anxiety | 1.023 | 1.018 | 1.028 | .003 | 79.182 | **<.001** |
| Touch recency x Attachment avoidance | .995 | .987 | 1.002 | .004 | 1.865 | .172 |
| Touch recency x Attachment anxiety | 1.006 | .999 | 1.013 | .003 | 2.888 | .089 |
| Touch satisfaction x Attachment avoidance | 1.002 | .995 | 1.009 | .004 | .280 | .597 |
| Touch satisfaction x Attachment anxiety | 1.004 | .998 | 1.011 | .003 | 1.849 | .174 |

| Table S19: Ordinal logistic regression predicting sleep latency with too much to just right touch satisfaction, including participants who experienced more recent touch. For gender, women are the reference category. N = 5199. | | | | | | |
| --- | --- | --- | --- | --- | --- | --- |
| **Sleep latency** | | | | | | |
|  |  | **95% Confidence Interval for OR** | |  |  |  |
| **Predictor** | **OR** | **Lower** | **Upper** | **SE** | ***χ*^2^** | ***p*** |
| Gender | .659 | .573 | .757 | .071 | 34.650 | **<.001** |
| Age | .994 | .990 | .998 | .002 | 10.416 | **.001** |
| End date | .998 | .995 | 1.001 | .001 | 2.186 | .139 |
| Loneliness | 1.016 | 1.009 | 1.023 | .004 | 19.407 | **<.001** |
| Childhood bed routine | .974 | .937 | 1.013 | .020 | 1.726 | .189 |
| Touch recency | 1.066 | .977 | 1.163 | .044 | 2.055 | .152 |
| Touch satisfaction - too much to just right | .908 | .745 | 1.107 | .101 | .909 | .340 |
| Attachment avoidance | 1.004 | .994 | 1.013 | .005 | .584 | .445 |
| Attachment anxiety | 1.027 | 1.019 | 1.035 | .004 | 40.619 | **<.001** |
| Touch recency x Attachment avoidance | .994 | .982 | 1.006 | .006 | 1.015 | .314 |
| Touch recency x Attachment anxiety | 1.008 | .997 | 1.018 | .005 | 2.058 | .151 |
| Touch satisfaction x Attachment avoidance | .978 | .957 | .999 | .011 | 4.105 | .043 |
| Touch satisfaction x Attachment anxiety | .997 | .975 | 1.020 | .011 | .059 | .808 |

| Table S20: Ordinal logistic regression predicting WASO number with too little to just right touch satisfaction, including participants who experienced more recent touch. For gender, women are the reference category. N = 10678. | | | | | | |
| --- | --- | --- | --- | --- | --- | --- |
| **WASO number** | | | | | | |
|  |  | **95% Confidence Interval for OR** | |  |  |  |
| **Predictor** | **OR** | **Lower** | **Upper** | **SE** | ***χ*^2^** | ***p*** |
| Gender | .907 | .835 | .987 | .043 | 5.185 | .023 |
| Age | 1.030 | 1.027 | 1.033 | .001 | 440.772 | **<.001** |
| End date | .995 | .993 | .997 | .001 | 22.179 | **<.001** |
| Loneliness | 1.011 | 1.007 | 1.015 | .002 | 25.157 | **<.001** |
| Childhood bed routine | 1.001 | .976 | 1.028 | .013 | .012 | .911 |
| Touch recency | .911 | .862 | .962 | .028 | 11.089 | **.001** |
| Touch satisfaction - too little to just right | .998 | .946 | 1.053 | .027 | .007 | .933 |
| Attachment avoidance | 1.003 | .997 | 1.009 | .003 | 1.159 | .282 |
| Attachment anxiety | 1.021 | 1.016 | 1.026 | .002 | 68.680 | **<.001** |
| Touch recency x Attachment avoidance | 1.006 | .998 | 1.014 | .004 | 2.275 | .131 |
| Touch recency x Attachment anxiety | .998 | .991 | 1.005 | .003 | .359 | .549 |
| Touch satisfaction x Attachment avoidance | .999 | .992 | 1.006 | .004 | .062 | .804 |
| Touch satisfaction x Attachment anxiety | 1.001 | .994 | 1.007 | .003 | .030 | .863 |

| Table S21: Ordinal logistic regression predicting WASO number with too much to just right touch satisfaction, including participants who experienced more recent touch. For gender, women are the reference category. N = 5200. | | | | | | |
| --- | --- | --- | --- | --- | --- | --- |
| **WASO number** | | | | | | |
|  |  | **95% Confidence Interval for OR** | |  |  |  |
| **Predictor** | **OR** | **Lower** | **Upper** | **SE** | ***χ*^2^** | ***p*** |
| Gender | .883 | .776 | 1.006 | .066 | 3.522 | .061 |
| Age | 1.026 | 1.022 | 1.030 | .002 | 175.337 | **<.001** |
| End date | .994 | .992 | .997 | .001 | 15.516 | **<.001** |
| Loneliness | 1.015 | 1.008 | 1.022 | .003 | 18.437 | **<.001** |
| Childhood bed routine | .965 | .929 | 1.002 | .019 | 3.380 | .066 |
| Touch recency | .893 | .821 | .972 | .043 | 6.887 | **.009** |
| Touch satisfaction - too much to just right | .714 | .586 | .869 | .100 | 11.233 | **.001** |
| Attachment avoidance | .999 | .990 | 1.008 | .005 | .038 | .845 |
| Attachment anxiety | 1.016 | 1.008 | 1.024 | .004 | 15.797 | **<.001** |
| Touch recency x Attachment avoidance | 1.003 | .992 | 1.014 | .006 | .259 | .611 |
| Touch recency x Attachment anxiety | .992 | .982 | 1.002 | .005 | 2.565 | .109 |
| Touch satisfaction x Attachment avoidance | .997 | .976 | 1.018 | .011 | .071 | .790 |
| Touch satisfaction x Attachment anxiety | .978 | .957 | .999 | .011 | 4.231 | .040 |

| Table S22: Ordinal logistic regression predicting WASO duration with too little to just right touch satisfaction, including participants who experienced more recent touch. For gender, women are the reference category. N = 10673. | | | | | | |
| --- | --- | --- | --- | --- | --- | --- |
| **WASO duration** | | | | | | |
|  |  | **95% Confidence Interval for OR** | |  |  |  |
| **Predictor** | **OR** | **Lower** | **Upper** | **SE** | ***χ*^2^** | ***p*** |
| Gender | .695 | .639 | .756 | .043 | 72.520 | **<.001** |
| Age | 1.026 | 1.023 | 1.029 | .001 | 345.875 | **<.001** |
| End date | .998 | .997 | 1.000 | .001 | 2.608 | .106 |
| Loneliness | 1.019 | 1.015 | 1.023 | .002 | 75.273 | **<.001** |
| Childhood bed routine | .978 | .953 | 1.004 | .013 | 2.844 | .092 |
| Touch recency | .909 | .861 | .959 | .028 | 11.950 | **.001** |
| Touch satisfaction - too little to just right | 1.036 | .983 | 1.093 | .027 | 1.738 | .187 |
| Attachment avoidance | 1.001 | .995 | 1.007 | .003 | .133 | .716 |
| Attachment anxiety | 1.016 | 1.011 | 1.021 | .002 | 41.852 | **<.001** |
| Touch recency x Attachment avoidance | .992 | .985 | 1.000 | .004 | 4.361 | .037 |
| Touch recency x Attachment anxiety | 1.002 | .995 | 1.008 | .003 | .337 | .562 |
| Touch satisfaction x Attachment avoidance | 1.001 | .994 | 1.008 | .004 | .138 | .710 |
| Touch satisfaction x Attachment anxiety | 1.001 | .995 | 1.007 | .003 | .061 | .805 |

| Table S23: Ordinal logistic regression predicting WASO duration with too much to just right touch satisfaction, including participants who experienced more recent touch. For gender, women are the reference category. N = 5197. | | | | | | |
| --- | --- | --- | --- | --- | --- | --- |
| **WASO duration** | | | | | | |
|  |  | **95% Confidence Interval for OR** | |  |  |  |
| **Predictor** | **OR** | **Lower** | **Upper** | **SE** | ***χ*^2^** | ***p*** |
| Gender | .703 | .618 | .800 | .066 | 28.504 | **<.001** |
| Age | 1.026 | 1.022 | 1.030 | .002 | 179.810 | **<.001** |
| End date | .998 | .995 | 1.001 | .001 | 1.998 | .157 |
| Loneliness | 1.022 | 1.015 | 1.029 | .003 | 41.300 | **<.001** |
| Childhood bed routine | .965 | .929 | 1.002 | .019 | 3.484 | .062 |
| Touch recency | .888 | .816 | .967 | .043 | 7.570 | **.006** |
| Touch satisfaction - too much to just right | .905 | .747 | 1.097 | .098 | 1.028 | .311 |
| Attachment avoidance | .999 | .990 | 1.008 | .005 | .069 | .793 |
| Attachment anxiety | 1.012 | 1.004 | 1.020 | .004 | 9.176 | **.002** |
| Touch recency x Attachment avoidance | .984 | .973 | .995 | .006 | 8.367 | **.004** |
| Touch recency x Attachment anxiety | 1.000 | .990 | 1.010 | .005 | .000 | .985 |
| Touch satisfaction x Attachment avoidance | 1.004 | .984 | 1.025 | .010 | .171 | .679 |
| Touch satisfaction x Attachment anxiety | 1.007 | .986 | 1.029 | .011 | .415 | .519 |

| Table S24: Ordinal logistic regression predicting sleep quality with too little to just right touch satisfaction, including participants who experienced less recent touch. For gender, women are the reference category. N = 940. | | | | | | |
| --- | --- | --- | --- | --- | --- | --- |
| **Sleep quality** | | | | | | |
|  |  | **95% Confidence Interval for OR** | |  |  |  |
| **Predictor** | **OR** | **Lower** | **Upper** | **SE** | ***χ*^2^** | ***p*** |
| Gender | .992 | .771 | 1.275 | .128 | .004 | .947 |
| Age | .991 | .981 | 1.000 | .005 | 3.480 | .062 |
| End date | .994 | .988 | 1.000 | .003 | 4.402 | .036 |
| Loneliness | 1.024 | 1.012 | 1.037 | .006 | 14.745 | **<.001** |
| Childhood bed routine | .939 | .864 | 1.021 | .043 | 2.186 | .139 |
| Touch recency | 1.038 | .835 | 1.289 | .111 | .111 | .739 |
| Touch satisfaction - too little to just right | 1.006 | .833 | 1.216 | .097 | .004 | .947 |
| Attachment avoidance | 1.001 | .948 | 1.057 | .028 | .001 | .976 |
| Attachment anxiety | 1.010 | .959 | 1.063 | .026 | .137 | .711 |
| Touch recency x Attachment avoidance | 1.002 | .981 | 1.022 | .010 | .021 | .885 |
| Touch recency x Attachment anxiety | 1.005 | .985 | 1.025 | .010 | .201 | .654 |
| Touch satisfaction x Attachment avoidance | 1.002 | .984 | 1.020 | .009 | .047 | .828 |
| Touch satisfaction x Attachment anxiety | 1.005 | .987 | 1.023 | .009 | .310 | .578 |

Sensitivity analyses for participants who experienced less recent touch.

| Table S25: Ordinal logistic regression predicting sleep quality with too much to just right touch satisfaction, including participants who experienced less recent touch. For gender, women are the reference category. N = 211. | | | | | | |
| --- | --- | --- | --- | --- | --- | --- |
| **Sleep quality** | | | | | | |
|  |  | **95% Confidence Interval for OR** | |  |  |  |
| **Predictor** | **OR** | **Lower** | **Upper** | **SE** | ***χ*^2^** | ***p*** |
| Gender | .876 | .497 | 1.547 | .290 | .207 | .649 |
| Age | 1.007 | .986 | 1.027 | .010 | .396 | .529 |
| End date | 1.000 | .987 | 1.012 | .007 | .003 | .954 |
| Loneliness | 1.013 | .986 | 1.040 | .014 | .886 | .347 |
| Childhood bed routine | .903 | .754 | 1.082 | .092 | 1.213 | .271 |
| Touch recency | 1.031 | .619 | 1.717 | .260 | .014 | .907 |
| Touch satisfaction - too much to just right | .984 | .377 | 2.569 | .489 | .001 | .974 |
| Attachment avoidance | 1.027 | .927 | 1.137 | .052 | .257 | .612 |
| Attachment anxiety | .979 | .880 | 1.090 | .054 | .145 | .704 |
| Touch recency x Attachment avoidance | .996 | .959 | 1.034 | .019 | .045 | .833 |
| Touch recency x Attachment anxiety | 1.022 | .980 | 1.065 | .021 | 1.016 | .313 |
| Touch satisfaction x Attachment avoidance | .954 | .892 | 1.021 | .034 | 1.850 | .174 |
| Touch satisfaction x Attachment anxiety | 1.019 | .967 | 1.074 | .027 | .491 | .484 |

| Table S26: Ordinal logistic regression predicting sleep duration with too little to just right touch satisfaction, including participants who experienced less recent touch. For gender, women are the reference category. N = 939. | | | | | | |
| --- | --- | --- | --- | --- | --- | --- |
| **Sleep duration** | | | | | | |
|  |  | **95% Confidence Interval for OR** | |  |  |  |
| **Predictor** | **OR** | **Lower** | **Upper** | **SE** | ***χ*^2^** | ***p*** |
| Gender | .926 | .725 | 1.184 | .125 | .375 | .540 |
| Age | .995 | .985 | 1.004 | .005 | 1.173 | .279 |
| End date | 1.000 | .995 | 1.006 | .003 | .015 | .903 |
| Loneliness | 1.021 | 1.009 | 1.033 | .006 | 11.692 | **.001** |
| Childhood bed routine | .960 | .886 | 1.040 | .041 | .999 | .317 |
| Touch recency | 1.096 | .891 | 1.347 | .105 | .753 | .386 |
| Touch satisfaction - too little to just right | 1.056 | .882 | 1.264 | .092 | .350 | .554 |
| Attachment avoidance | 1.009 | .958 | 1.062 | .026 | .107 | .743 |
| Attachment anxiety | 1.025 | .977 | 1.077 | .025 | 1.015 | .314 |
| Touch recency x Attachment avoidance | 1.001 | .981 | 1.020 | .010 | .005 | .946 |
| Touch recency x Attachment anxiety | .991 | .972 | 1.009 | .010 | .957 | .328 |
| Touch satisfaction x Attachment avoidance | .992 | .975 | 1.010 | .009 | .776 | .378 |
| Touch satisfaction x Attachment anxiety | 1.003 | .986 | 1.021 | .009 | .125 | .723 |

| Table S27: Ordinal logistic regression predicting sleep duration with too much to just right touch satisfaction, including participants who experienced less recent touch. For gender, women are the reference category. N = 210. | | | | | | |
| --- | --- | --- | --- | --- | --- | --- |
| **Sleep duration** | | | | | | |
|  |  | **95% Confidence Interval for OR** | |  |  |  |
| **Predictor** | **OR** | **Lower** | **Upper** | **SE** | ***χ*^2^** | ***p*** |
| Gender | 1.023 | .586 | 1.785 | .284 | .006 | .937 |
| Age | .988 | .969 | 1.007 | .010 | 1.537 | .215 |
| End date | 1.003 | .991 | 1.016 | .006 | .260 | .610 |
| Loneliness | 1.007 | .981 | 1.033 | .013 | .243 | .622 |
| Childhood bed routine | .896 | .749 | 1.072 | .092 | 1.444 | .230 |
| Touch recency | 1.126 | .681 | 1.861 | .256 | .215 | .643 |
| Touch satisfaction - too much to just right | 2.717 | 1.095 | 6.741 | .464 | 4.647 | .031 |
| Attachment avoidance | 1.016 | .916 | 1.127 | .053 | .093 | .761 |
| Attachment anxiety | .991 | .889 | 1.104 | .055 | .030 | .863 |
| Touch recency x Attachment avoidance | .999 | .961 | 1.038 | .020 | .005 | .942 |
| Touch recency x Attachment anxiety | 1.008 | .967 | 1.051 | .021 | .143 | .705 |
| Touch satisfaction x Attachment avoidance | .870 | .808 | .938 | .038 | 13.244 | **<.001** |
| Touch satisfaction x Attachment anxiety | 1.023 | .967 | 1.082 | .029 | .618 | .432 |

| Table S28: Ordinal logistic regression predicting sleep latency with too little to just right touch satisfaction, including participants who experienced less recent touch. For gender, women are the reference category. N = 939. | | | | | | |
| --- | --- | --- | --- | --- | --- | --- |
| **Sleep latency** | | | | | | |
|  |  | **95% Confidence Interval for OR** | |  |  |  |
| **Predictor** | **OR** | **Lower** | **Upper** | **SE** | ***χ*^2^** | ***p*** |
| Gender | .528 | .409 | .682 | .131 | 23.890 | **<.001** |
| Age | .994 | .984 | 1.003 | .005 | 1.729 | .189 |
| End date | .997 | .992 | 1.003 | .003 | .715 | .398 |
| Loneliness | 1.016 | 1.004 | 1.029 | .006 | 6.849 | **.009** |
| Childhood bed routine | 1.007 | .927 | 1.094 | .042 | .025 | .874 |
| Touch recency | 1.053 | .849 | 1.307 | .110 | .223 | .636 |
| Touch satisfaction - too little to just right | 1.094 | .912 | 1.314 | .093 | .935 | .334 |
| Attachment avoidance | .983 | .932 | 1.037 | .027 | .388 | .534 |
| Attachment anxiety | .987 | .939 | 1.038 | .026 | .261 | .609 |
| Touch recency x Attachment avoidance | 1.009 | .989 | 1.029 | .010 | .708 | .400 |
| Touch recency x Attachment anxiety | 1.010 | .990 | 1.030 | .010 | .984 | .321 |
| Touch satisfaction x Attachment avoidance | 1.013 | .995 | 1.031 | .009 | 1.962 | .161 |
| Touch satisfaction x Attachment anxiety | 1.008 | .990 | 1.025 | .009 | .726 | .394 |

| Table S29: Ordinal logistic regression predicting sleep latency with too much to just right touch satisfaction, including participants who experienced less recent touch. For gender, women are the reference category. N = 210. | | | | | | |
| --- | --- | --- | --- | --- | --- | --- |
| **Sleep latency** | | | | | | |
|  |  | **95% Confidence Interval for OR** | |  |  |  |
| **Predictor** | **OR** | **Lower** | **Upper** | **SE** | ***χ*^2^** | ***p*** |
| Gender | .653 | .366 | 1.165 | .295 | 2.083 | .149 |
| Age | .994 | .974 | 1.014 | .010 | .318 | .573 |
| End date | .998 | .985 | 1.011 | .007 | .094 | .759 |
| Loneliness | 1.022 | .996 | 1.048 | .013 | 2.643 | .104 |
| Childhood bed routine | .989 | .822 | 1.190 | .094 | .014 | .905 |
| Touch recency | .739 | .440 | 1.241 | .264 | 1.305 | .253 |
| Touch satisfaction - too much to just right | 4.175 | 1.451 | 12.009 | .539 | 7.026 | **.008** |
| Attachment avoidance | 1.012 | .910 | 1.125 | .054 | .046 | .830 |
| Attachment anxiety | .971 | .869 | 1.084 | .056 | .281 | .596 |
| Touch recency x Attachment avoidance | 1.006 | .966 | 1.047 | .021 | .076 | .783 |
| Touch recency x Attachment anxiety | 1.020 | .976 | 1.066 | .023 | .798 | .372 |
| Touch satisfaction x Attachment avoidance | .887 | .822 | .958 | .039 | 9.327 | **.002** |
| Touch satisfaction x Attachment anxiety | 1.028 | .971 | 1.089 | .029 | .899 | .343 |

| Table S30: Ordinal logistic regression predicting WASO number with too little to just right touch satisfaction, including participants who experienced less recent touch. For gender, women are the reference category. N = 939. | | | | | | |
| --- | --- | --- | --- | --- | --- | --- |
| **WASO number** | | | | | | |
|  |  | | **95% Confidence** | | |  |
|  |  |  | **Interval for OR** | | |  |
| **Predictor** | **OR** | **Lower** | | **Upper** | **SE** | |
| Gender | .801 | .627 | 1.024 | .125 | 3.139 | .076 |
| Age | 1.029 | 1.019 | 1.039 | .005 | 32.230 | **<.001** |
| End date | .999 | .993 | 1.005 | .003 | .087 | .768 |
| Loneliness | 1.007 | .995 | 1.019 | .006 | 1.415 | .234 |
| Childhood bed routine | .979 | .902 | 1.062 | .042 | .267 | .606 |
| Touch recency | 1.094 | .889 | 1.347 | .106 | .727 | .394 |
| Touch satisfaction - too little to just right | 1.010 | .840 | 1.216 | .095 | .012 | .913 |
| Attachment avoidance | .986 | .935 | 1.039 | .027 | .276 | .599 |
| Attachment anxiety | .997 | .948 | 1.048 | .025 | .017 | .897 |
| Touch recency x Attachment avoidance | 1.012 | .992 | 1.032 | .010 | 1.361 | .243 |
| Touch recency x Attachment anxiety | 1.004 | .985 | 1.024 | .010 | .187 | .666 |
| Touch satisfaction x Attachment avoidance | .993 | .976 | 1.011 | .009 | .536 | .464 |
| Touch satisfaction x Attachment anxiety | 1.003 | .986 | 1.022 | .009 | .145 | .703 |

| Table S31: Ordinal logistic regression predicting WASO number with too much to just right touch satisfaction, including participants who experienced less recent touch. For gender, women are the reference category. N = 210. | | | | | | |
| --- | --- | --- | --- | --- | --- | --- |
| **WASO number** | | | | | | |
|  |  | | **95% Confidence** | | |  |
|  |  |  | **Interval for OR** | | |  |
| **Predictor** | **OR** | **Lower** | | **Upper** | **SE** | |
| Gender | .848 | .488 | 1.474 | .282 | .341 | .559 |
| Age | 1.034 | 1.013 | 1.056 | .010 | 10.244 | **.001** |
| End date | .994 | .981 | 1.006 | .006 | 1.028 | .311 |
| Loneliness | 1.001 | .976 | 1.026 | .013 | .005 | .941 |
| Childhood bed routine | .955 | .801 | 1.139 | .090 | .262 | .609 |
| Touch recency | 1.248 | .759 | 2.051 | .253 | .764 | .382 |
| Touch satisfaction - too much to just right | 1.131 | .447 | 2.864 | .474 | .068 | .795 |
| Attachment avoidance | 1.053 | .954 | 1.162 | .050 | 1.052 | .305 |
| Attachment anxiety | 1.028 | .920 | 1.148 | .056 | .238 | .625 |
| Touch recency x Attachment avoidance | .986 | .950 | 1.024 | .019 | .540 | .462 |
| Touch recency x Attachment anxiety | .996 | .953 | 1.041 | .023 | .032 | .857 |
| Touch satisfaction x Attachment avoidance | .937 | .872 | 1.006 | .036 | 3.263 | .071 |
| Touch satisfaction x Attachment anxiety | 1.012 | .959 | 1.067 | .027 | .185 | .667 |

| Table S32: Ordinal logistic regression predicting WASO duration with too little to just right touch satisfaction, including participants who experienced less recent touch. For gender, women are the reference category. N = 937. | | | | | | |
| --- | --- | --- | --- | --- | --- | --- |
| **WASO duration** | | | | | | |
|  |  | **95% Confidence Interval for OR** | |  |  |  |
| **Predictor** | **OR** | **Lower** | **Upper** | **SE** | ***χ*^2^** | **p** |
| Gender | .730 | .572 | .932 | .125 | 6.362 | .012 |
| Age | 1.019 | 1.009 | 1.029 | .005 | 14.373 | **<.001** |
| End date | 1.002 | .996 | 1.007 | .003 | .271 | .603 |
| Loneliness | 1.008 | .996 | 1.020 | .006 | 1.743 | .187 |
| Childhood bed routine | .965 | .890 | 1.046 | .041 | .749 | .387 |
| Touch recency | 1.072 | .872 | 1.317 | .105 | .437 | .508 |
| Touch satisfaction - too little to just right | 1.173 | .977 | 1.408 | .093 | 2.929 | .087 |
| Attachment avoidance | 1.005 | .954 | 1.058 | .027 | .029 | .864 |
| Attachment anxiety | .999 | .951 | 1.048 | .025 | .002 | .960 |
| Touch recency x Attachment avoidance | .998 | .979 | 1.018 | .010 | .030 | .862 |
| Touch recency x Attachment anxiety | 1.001 | .982 | 1.020 | .010 | .006 | .937 |
| Touch satisfaction x Attachment avoidance | .984 | .967 | 1.002 | .009 | 3.130 | .077 |
| Touch satisfaction x Attachment anxiety | .998 | .981 | 1.015 | .009 | .057 | .811 |

| Table S33: Ordinal logistic regression predicting WASO duration with too much to just right touch satisfaction, including participants who experienced less recent touch. For gender, women are the reference category. N = 210. | | | | | | |
| --- | --- | --- | --- | --- | --- | --- |
| **WASO duration** | | | | | | |
|  |  | **95% Confidence Interval for OR** | |  |  |  |
| **Predictor** | **OR** | **Lower** | **Upper** | **SE** | ***χ*^2^** | **p** |
| Gender | .665 | .383 | 1.152 | .281 | 2.117 | .146 |
| Age | 1.025 | 1.005 | 1.046 | .010 | 5.883 | .015 |
| End date | 1.002 | .990 | 1.015 | .006 | .096 | .757 |
| Loneliness | 1.008 | .983 | 1.034 | .013 | .413 | .521 |
| Childhood bed routine | .960 | .802 | 1.148 | .092 | .201 | .654 |
| Touch recency | .927 | .562 | 1.531 | .256 | .087 | .768 |
| Touch satisfaction - too much to just right | 2.694 | 1.000 | 7.252 | .505 | 3.845 | .050 |
| Attachment avoidance | 1.042 | .942 | 1.152 | .051 | .633 | .426 |
| Attachment anxiety | 1.020 | .918 | 1.134 | .054 | .140 | .709 |
| Touch recency x Attachment avoidance | .982 | .946 | 1.020 | .019 | .839 | .360 |
| Touch recency x Attachment anxiety | .992 | .951 | 1.035 | .022 | .141 | .707 |
| Touch satisfaction x Attachment avoidance | .909 | .846 | .976 | .036 | 6.923 | **.009** |
| Touch satisfaction x Attachment anxiety | 1.004 | .951 | 1.060 | .028 | .018 | .894 |
